# Cytotoxic and Antioxidant Activities of Imine Analogs of Trans-Resveratrol towards Murine Neuronal N2a Cells

**DOI:** 10.3390/molecules27154713

**Published:** 2022-07-23

**Authors:** Mohamed Ksila, Anne Vejux, Emmanuelle Prost-Camus, Philippe Durand, Imen Ghzaiel, Thomas Nury, Dorian Duprey, Smail Meziane, Olfa Masmoudi-Kouki, Norbert Latruffe, Taoufik Ghrairi, Michel Prost, Gérard Lizard, Dominique Vervandier-Fasseur

**Affiliations:** 1 Team ‘Biochemistry of the Peroxisome, Inflammation and Lipid Metabolism’ EA7270/Inserm, University Bourgogne Franche-Comté, 21000 Dijon, France; mohamedksila44@gmail.com (M.K.); anne.vejux@u-bourgogne.fr (A.V.); imenghzaiel93@gmail.com (I.G.); thomas.nury@u-bourgogne.fr (T.N.); norbert.latruffe@u-bourgogne.fr (N.L.); 2 Laboratory of Neurophysiology, Cellular Physiopathology and Valorisation of Biomolecules, (LR18ES03), Department of Biology, Faculty of Sciences, University Tunis El Manar, Tunis 2092, Tunisia; olfa.masmoudi@fst.utm.tn (O.M.-K.); taoufik.ghrairi@fst.utm.tn (T.G.); 3 Laboratoire Spiral, 21560 Couternon, France; camusprost@hotmail.fr (E.P.-C.); laraspiral@wanadoo.fr (P.D.); michelprost.spiral@wanadoo.fr (M.P.); 4 Institut Européen des Antioxydants (IEA), 1B, rue Victor de Lespinats, 54230 Neuves-Maisons, France; smeziane@ie-antioxydants.com; 5 Team OCS, Institute of Molecular Chemistry of University of Burgundy (ICMUB UMR CNRS 6302), University of Bourgogne Franche-Comté, 21000 Dijon, France; Dorian_Duprey@etu.u-bourgogne.fr

**Keywords:** *trans-*resveratrol, aza-stilbenes synthesis, antioxidant activity, cytotoxicity, murine neuronal N2a cells

## Abstract

*Trans*-resveratrol is a natural polyphenol showing numerous biological properties, especially anti-tumoral and antioxidant activity. Among numerous resveratrol derivatives, aza-stilbenes, which bear an imine bound, show interesting biological activities. In the present study, we synthesized a series of imine analogs of *trans*-resveratrol (seven aza-stilbenes) following an easy and low-cost procedure of green chemistry. The toxicity of synthesized aza-stilbenes, which is currently unknown, was evaluated on murine neuronal N2a cells, comparatively to *trans*-resveratrol, by considering: cell density evaluated by staining with sulforhodamine 101; esterase activity, which is a criteria of cell viability, by staining with fluorescein diacetate; and transmembrane mitochondrial potential, which is known to decrease during cell death, by staining with DiOC_6_(3) using flow cytometry. In addition, the antioxidant activity was quantified with the KRL (Kit Radicaux Libres) assay, the DPPH (2,2′-diphenyl-1-picrylhydrazyl radical) assay and the FRAP (ferric reducing antioxidant power) assay. The PAOT (Pouvoir Antioxidant Total) score was also used. The aza-stilbenes provide different cytotoxic and antioxidant activities, which are either higher or lower than those of *trans*-resveratrol. Based on their cytotoxic and antioxidant characteristics, all synthesized aza-stilbenes are distinguished from *trans*-resveratrol.

## 1. Introduction

Polyphenolic compounds present in numerous plants exhibit a large variety of biological properties [1]. Among them, polyphenolic stilbenoids occupy an important place in the field of health-beneficial molecules. In this series, the most-studied molecule is the *trans*-resveratrol (RSV) or 3,4′,5-trihydroxystilbene, a phytoalexin, present in numerous edible plants such as peanuts, red fruit including grapes and therefore in red wine.

**Figure 1:**
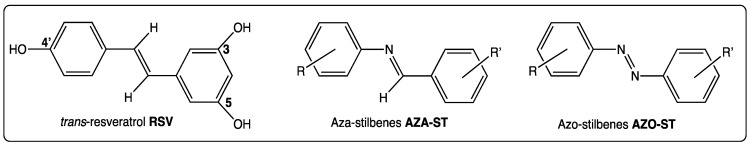
Structure of *trans*-resveratrol **RSV**, aza-stilbenes **AZA-ST** and azo-stilbenes **AZO-ST**.

RSV is implied in the French paradox [2,3] and in the Mediterranean diet [4,5]. The interest aroused by RSV since the 1990s [6] is especially due to its pleiotropic antifungal and therapeutic activities in the treatment of inflammatory diseases and some cancer. Unfortunately, the weak bioavailability of RSV does not allow to consider it as a perfect therapeutic molecule [7]. Indeed, RSV is rapidly metabolized and degraded [5]. Numerous synthetic derivatives of RSV were elaborated to improve and target more of some biological activities and to enhance their water solubility. Different ways to modify RSV while keeping the initial stilbenoid skeleton were reported as transformation of phenolic functions into ester or ether functions [8,9,10], or by addition of different substituents on the aromatic rings [11,12,13,14], or by changing a phenyl with an aromatic heterocycle [15] or an aromatic organometallic cycle [13]. Moreover, the C = C bond may be replaced with an isosteric fragment as an aromatic heterocycle [16], or with a C = N bond or a N = N bond to provide aza-stilbenes **AZA-ST** [17] and azo-stilbenes **AZO-ST [18]**, respectively (Figure 1).

**Figure 2:**
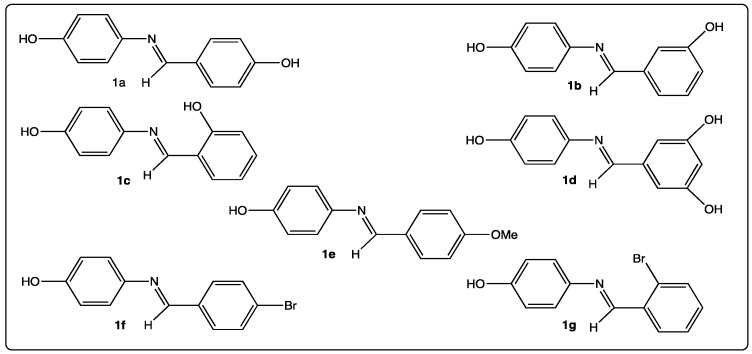
Structure of aza-stilbenes **1a**–**1g**.

Recently, biological activities of these series of bio-isosteric resveratrol derivatives were reviewed, especially the antioxidant properties of **AZA-ST [19]**. Regarding resveratrol, the replacement of the C = C bond by the isosteric fragment C = N gives noteworthy behaviors to these derivatives. In addition, the presence of one or more hydroxyl groups and their position on aromatic cycles bring more or less strong antioxidant properties to aza-stilbenes. In this report, seven aza-stilbenes **1a**–**1g** were synthesized by using previously described methods, which were improved by including criteria of green chemistry [20,21,22,23,24,25] (Figure 2), and we evaluated their toxicity on murine neuronal N2a cells, comparatively to *trans*-resveratrol, by taking into account the following parameters: cell density evaluated by staining with sulforhodamine 101 (SR101); esterase activity, which is a criteria of cell viability, by staining with fluorescein diacetate (FDA); transmembrane mitochondrial potential (ΔΨm), which is known to decrease during cell death, by staining with 3,3′-dihexyloxacarbocyanine Iodide (DiOC_6_(3)) and flow cytometry; antioxidant activity was quantified with the KRL (Kit Radicaux Libres) assay as previously described [26,27] as well as with the DPPH (2,2′-diphenyl-1-picrylhydrazyl radical) assay [28] and the ferric reducing antioxidant power FRAP (ferric reducing antioxidant power) assay [29,30]. The PAOT (Pouvoir Antioxydant Total) score was also used [31].

As it is assumed that aza-stilbenes would be less degraded than *trans-*resveratrol both in vitro and in vivo, their use for therapeutic purposes could be considered, provided that these molecules do not exhibit a greater toxicity than that of *trans-*resveratrol while having retained the antioxidant properties of this one.

## 2. Results and Discussion

### 2.1. Chemistry

**Scheme 1:**
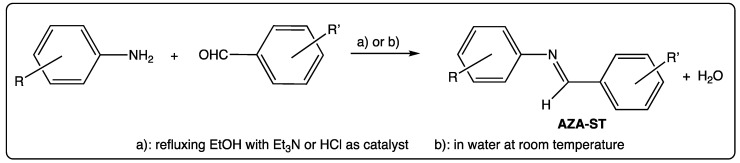
Synthetic methods for obtaining aza-stilbenes **AZA-ST**.

Aza-stilbenes may be easily synthesized by a one-step condensation reaction between aromatic aldehydes and primary aromatic amines (Scheme 1). The commercially available reactants often bear hydroxyl, methoxyl, methyl or halogenated atoms as substituents, which makes it possible to obtain a large series of aza-stilbenes. The reaction between an equimolar amount of aromatic aldehydes and primary aromatic amines may be carried out in refluxing EtOH with a catalytic amount of triethylamine [20] or of HCl [21,22]. However, aza-stilbenes may be obtained by stirring an equimolar amount of aromatic aldehydes and primary aromatic amines in a small volume of water as solvent at room temperature during 0.5 to 3 h. The products are isolated by filtration with high yields [23]. In this report, we chose this cost-effective method [24,25] that we have improved by introducing several criteria of green chemistry.

**Scheme 2:**
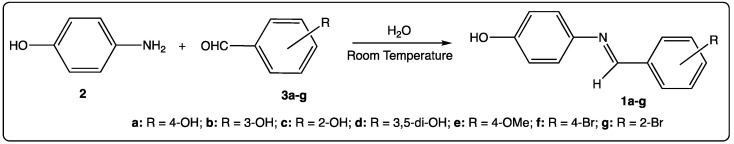
Synthesis of AZA-ST **1a**–**g**.

It has been shown that the antioxidant activities of *trans*-resveratrol (further defined as resveratrol) are often due to an OH group in position 4′ [32,33]. Thus, to keep a comparison setting between resveratrol and aza-stilbenes, **AZA-ST 1a**–**g** were prepared from 4-hydroxyaniline (**2**) and different aromatic aldehydes **3a-g** as described in Scheme 2. Compounds **1a**–**1e** have been reported by different authors [20,21]. Among them, Kotora et al. have characterized aza-stilbenes **1a**–**d,** especially by NMR spectroscopy [25]. In this report, we have carried out ^1^H, COSY and NOESY NMR experiments of all compounds to assign chemical shifts of aromatic protons as well as ^13^C, jmod, HSQC and HMBC NMR experiments to highlight coupling between the ^1^H and ^13^C nucleus.

Towards stilbenes synthesis, preparation of **AZA-ST** may be carried out in a one-step sustainable reaction. Indeed, synthesis of hydroxystilbenes derived from resveratrol requires several steps, including protection and deprotection reactions [11,13]. In addition, stilbenes are isolated under a mixture of both *E* and *Z* isomers, whose proportions depend on the method used (Heck or Wittig method) [13]. In contrast, in the case of **AZA-ST**, only the *E* isomers are isolated because the Z configuration is not thermally favored [34].

### 2.2. Cytotoxicity

**Figure 3:**
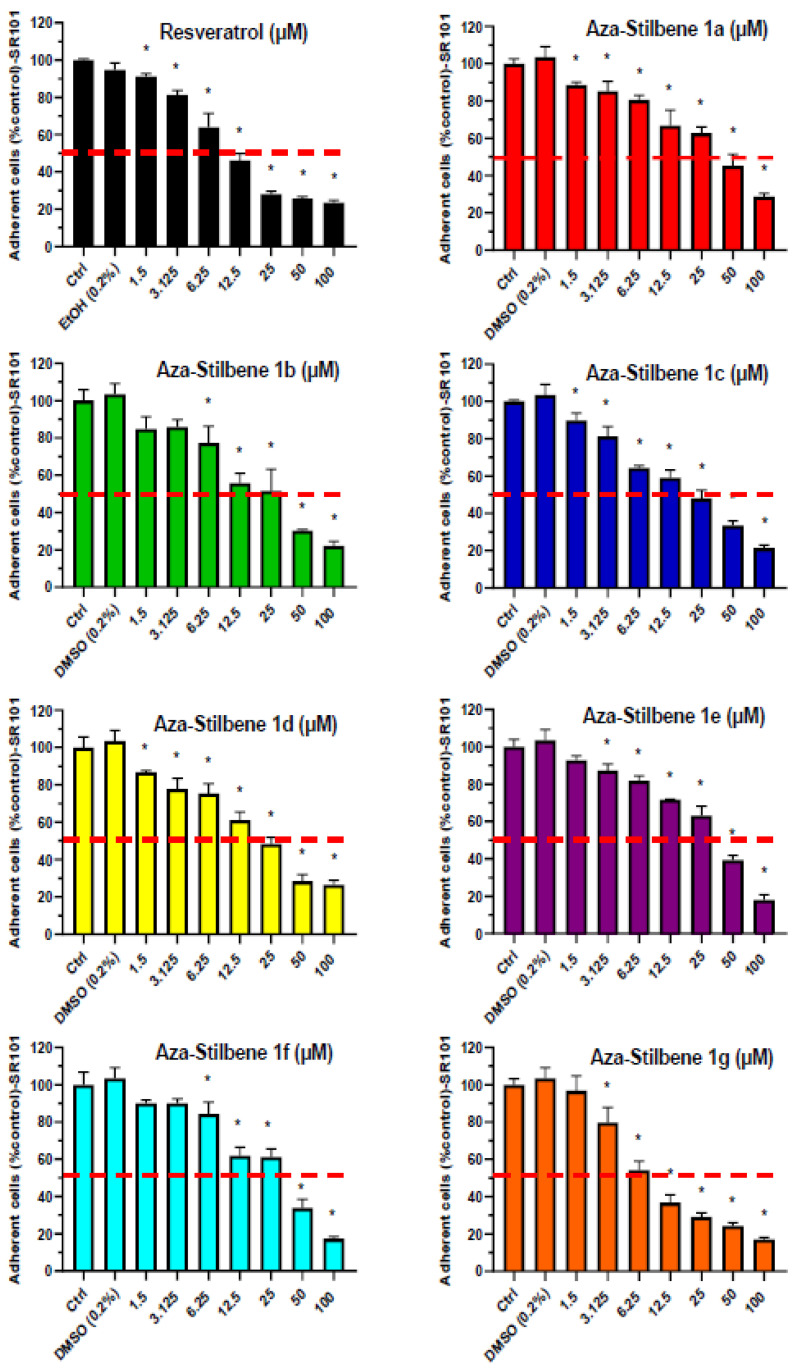
Evaluation with the sulforhodamine 101 (SR101) assay of the effects of resveratrol and aza-stilbenes **1a** to **1g** on cell density. N2a cells were incubated for 48 h with or without resveratrol or aza-stilbenes **1a** to **1g** in a range of concentrations from 1.5 to 100 µM. Cell density evaluated with SR101 reflects the quantity of adherent cells. The dotted red line makes it possible to evaluate the value of the concentration or the range of concentrations reducing the number of adherent cells by 50% (IC50). Data are the mean ± SD of two independent experiments performed in triplicate. Significance of the differences between control (untreated cells), Resveratrol (*trans-*resveratrol) or aza-stilbenes **1a** to **1g**–treated cells; Student’s *t* test: * *p* < 0.05 or less.

Some experiments were realized to determine and compare the cytotoxic activities of aza-stilbenes **1a** to **1g** to resveratrol in a range of concentration from 1.5 to 100 µM (48 h). In this range of concentration, resveratrol is known to have differentiating activities on N2a cells at low concentrations (6.25 and 12.5 µM, 48 h) [35] and to induce cell death at concentrations higher than 12.5 µM [36]. In the present study, three complementary assays (SR101, FDA and DiOC_6_(3) assays) were used to determine the cytotoxic activities of aza-stilbenes **1a** to **1g** comparatively to resveratrol. Among these different assays, SR101 gives information on cell density, which reflects cell growth and cell adhesion. With the different aza-stilbenes used, as well as with resveratrol, the decrease of fluorescence observed with the SR101 assay corresponds to a decrease of cell density, which is relied with a loss of cell adhesion associated with an increasing of floating cells (dead cells) in the culture medium (Figure 3).

**Figure 4:**
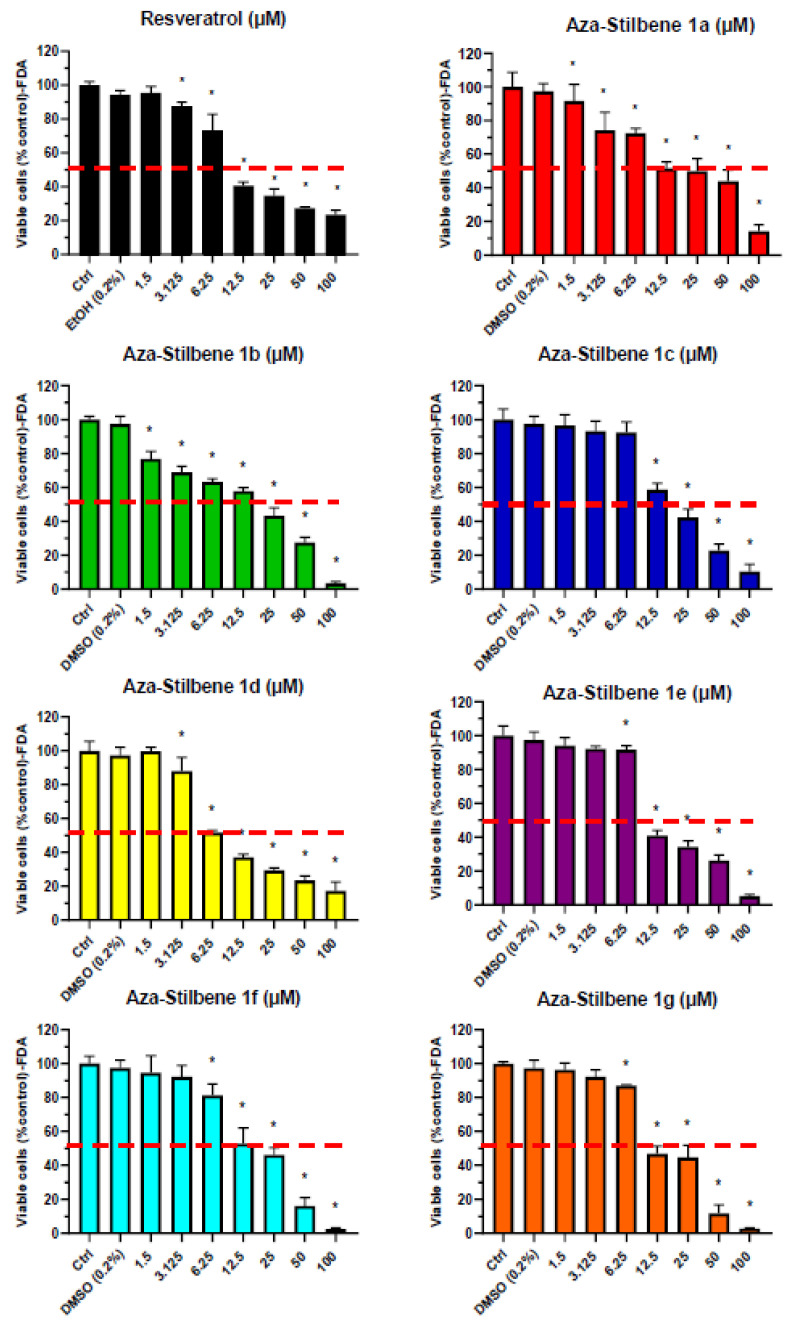
Evaluation with the fluorescein diacetate (FDA) assay of the effects of resveratrol and aza-stilbenes **1a** to **1g** on cell viability. N2a cells were incubated for 48 h with or without resveratrol or aza-stilbenes **1a** to **1g** in a range of concentrations from 1.5 to 100 µM. The dotted red line makes it possible to evaluate the value of the concentration, or the range of concentrations, reducing cell viability by 50% (IC50). Data are the mean ± SD of two independent experiments performed in triplicate. Significance of the differences between control (untreated cells), Resveratrol (*trans*-resveratrol) or aza-stilbenes **1a** to **1g**–treated cells; Student’s *t* test: * *p* < 0.05 or less.

Based on the SR101 test, the cytotoxicity of aza-stilbenes and resveratrol were in the following range of order: resveratrol > aza-stilbene **1g** > aza-stilbene **1b** > aza-stilbene **1d** > aza-stilbene **1c** >aza-stilbene **1a** > aza-stilbene **1d** > aza-stilbene **1e** (Figure 3). The FDA assay indicates a loss of esterase activity, obtained in the same range of concentrations as the loss of cell adhesion observed with SR101 (Figure 4). This supports that aza-stilbenes- and resveratrol-induced cell death trigger plasma-membrane damages; this agrees with the concentration-dependent decrease of esterase activity observed with the different aza-stilbenes studied and with resveratrol.

**Figure 5:**
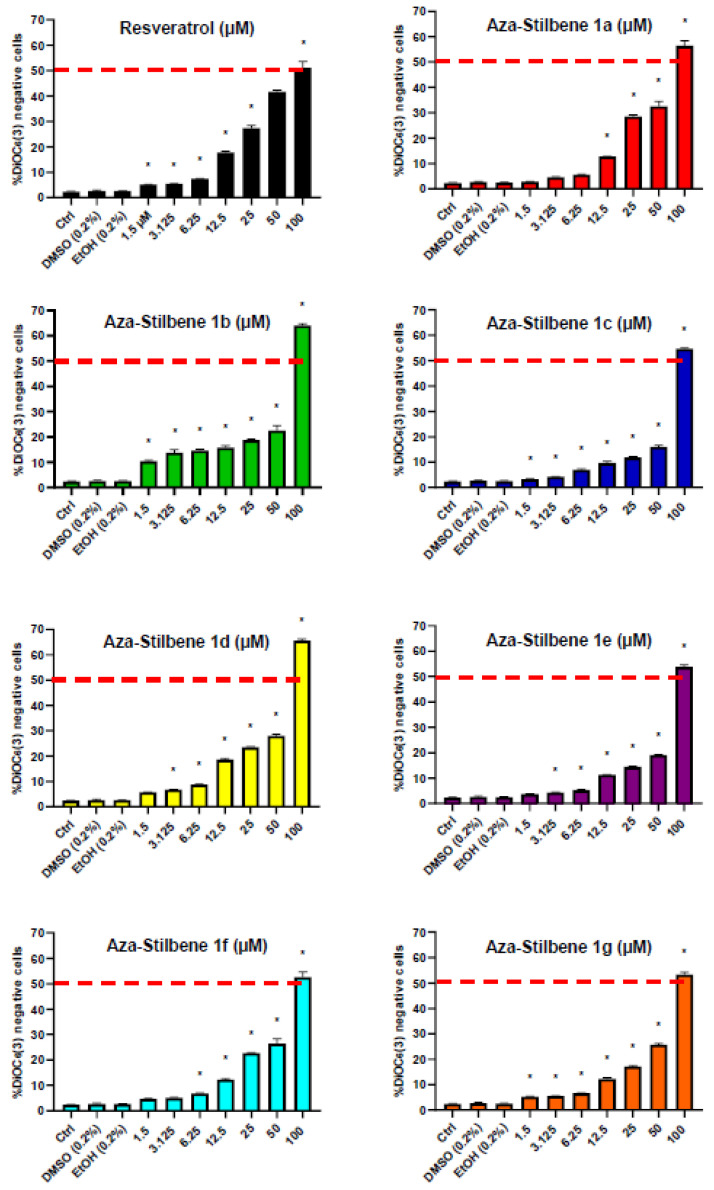
Flow cytometric evaluation of transmembrane mitochondrial potential (ΔΨm) with DiOC6(3) under treatment with resveratrol and aza-stilbenes **1a** to **1g**. N2a cells were incubated for 48 h with or without resveratrol or aza-stilbenes **1a** to **1g** in a range of concentrations from 1.5 to 100 µM. Data are the mean ± SD of two independent experiments performed in triplicate. Significance of the differences between control (untreated cells), Resveratrol (*trans*-resveratrol) or aza-stilbenes **1a** to **1g**–treated cells; Student’s *t* test: * *p* < 0.05 or less.

As it is well established on different cell types, including N2a cells, that resveratrol triggers mitochondrial dysfunctions in a concentration-dependent manner [36], the effects of aza-stilbenes have been studied at the mitochondrial level and compared with those of resveratrol. With the used of DiOC_6_(3), which allows quantifying the ΔΨm, a more or less pronounced loss of ΔΨm was observed with the different aza-stilbenes since 12.5 µM (Figure 5).

Our data show important differences of toxicity from one aza-stilbene to another. In addition, based on the different cytotoxic assays used, the most cytotoxic aza-stilbenes are aza-stilbene **1g** and aza-stilbene **1b** (Table 1). The toxicity of these two aza-stilbenes evocates the toxicity observed with resveratrol, whereas the toxicities of the other aza-stilbenes (**1a, 1c**, **1d**, **1e** and **1f**) are lower than with resveratrol.

**Figure 6:**
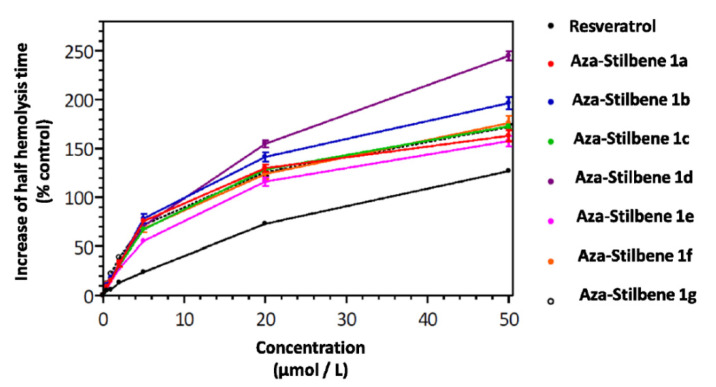
Quantification of the antioxidant activity of resveratrol and aza-stilbenes **1a** to **1g** with the KRL test.

In addition, as resveratrol is also known for its antioxidant properties, the antioxidant activities of aza-stilbenes **1a** to **1g** were determined with the KRL assay and compared with resveratrol. All the aza-stilbenes considered have higher antioxidant activities than resveratrol (Figure 6). The highest antioxidant activities were observed with aza-stilbenes **1d** and **1b**; the antioxidant activities of aza-stilbenes (**1a**, **1c**, **1e**, **1f** and **1g**) were similar and lower (Figure 6).

Data obtained with the KRL assay, which integrates the ability of an antioxidant molecule to neutralize reactive oxygen species and to prevent the peroxidation of membrane lipids, were associated with two conventional antioxidant assays: the DPPH (2,2′-diphenyl-1-picrylhydrazyl radical) and the FRAP (ferric reducing antioxidant power) assays. The data obtained with the DPPH assay, which only take in consideration the ability of an antioxidant molecule to neutralize a radical, allow to distinguish the different aza-stilbenes and the resveratrol. With the DPPH assay (at the exception of aza-stilbene **1c** and **1g**), as observed with the KRL test, the aza-stilbenes have higher antioxidant activities than resveratrol (Table 2).

As polyphenols are known for their chelating properties towards various metals, including Fe [37], it is supposed that the antioxidant and chelating properties of polyphenols, and probably of aza-stilbenes, could interfere when the FRAP assay is used. For this reason, in agreement with this hypothesis, it was unable to discriminate the antioxidant properties of resveratrol and aza-stilbenes with this method.

The PAOT score, which has been recently described [25], was also used and compared with the KRL and DPPH assay. The PAOT score is based on the measurement of the scavenging properties of antioxidants with the use of an electrochemical method. The data obtained with resveratrol and few aza-stilbenes also allow to distinguish the molecules tested. Based on the simultaneous use of the KRL, DPPH and PAOT assay, it is considered that the aza-stilbenes 1a and 1d have the highest antioxidant activities, whereas aza-stilbene 1c has the lowest antioxidant characteristics (Table 1).

It is important to underline that some differences were observed from one antioxidant assay to another, since the different assays used to evaluate the antioxidant activities measure different parameters.

As aza-stilbenes have antioxidant activities, it will be further of interest to evaluate their differentiating activities on N2a cells to determine their neurotrophic effect (antioxidant activity + differentiating activity on nerve cells) [35,38]. This can have important applications in the context of regenerative medicine. In addition, as resveratrol has also been shown to induce the differentiation of murine myoblasts C2C12 in myotubes [39], there is also an interest to further evaluate the ability of aza-stilbenes to act on the differentiation of skeletal muscle cells. This can also have important applications in the context of aging for the treatment of sarcopenia, which is characterized by a decrease of the muscular mass in the elderly [40].

## 3. Conclusions

Comparatively to resveratrol (*trans*-resveratrol), the aza-stilbenes synthetized have either higher or lower cytotoxic and antioxidant activities. Our data show that all aza-stilbenes synthesized are distinguishable based on their cytotoxic and antioxidant activities. Thus, the aza-stilbenes produced constitute a new series of molecules for which it is therefore justified to further specify their pharmacological activities on different in vitro and in vivo disease models for which the *trans-*resveratrol has shown some effects such as some cancer, chronic inflammatory diseases and age-related diseases (cardiovascular diseases, ocular diseases and neurodegenerative diseases).

## 4. Materials and Methods

### 4.1. Chemistry

All reagents and solvents are purchased from commercial suppliers and used without further purifications. 4-aminophenol [123-30-8], 3-hydroxybenzaldehyde [100-83-4], salicylaldehyde [90-02-8] and 3,5-dihydroxybenzaldehyde [26153-38-8] are purchased from Alfa Aesar (ThermoFisher Scientific, Waltham, MA USA); 4-hydroxybenzaldehyde [123-08-0] and *para*-anisaldehyde [123-11-5] are purchased from Acros Organics (Geel/Antwerp, Belgium); 2-bromobenzaldehyde [6630-33-7] was purchased from Aldrich and 4-bromobenzaldehyde [1122-91-4] is purchased from TCI Europe (Zwijndrecht, Belgium).

**Figure 7:**
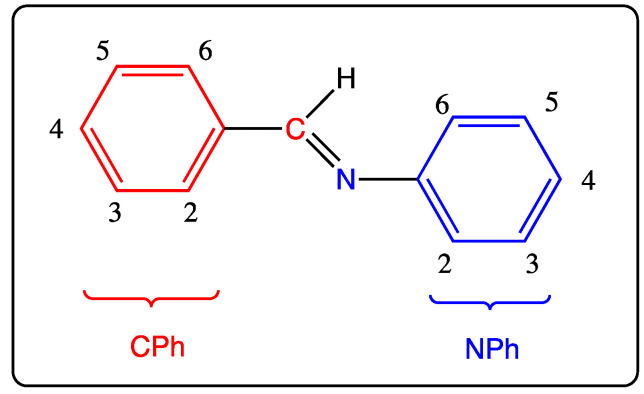
Symbolism for analysis of NMR data.

The characterization of the products was established at the “Chemical Analysis Platform of Molecular Synthesis University of Burgundy (PACSMUB)”. High-resolution mass spectra (HRMS) were obtained on a Thermo LTQ-Orbitrap XL with ESI source. FTIR spectra were obtained on a Brucker Alfa spectrometer (diamond ATR, Kontich, Belgium) in the range of 400–4000 cm^−1^. ^1^H (500 MHz) and ^13^C (126 MHz) NMR spectra were recorded on Brucker 500 MHz spectrometer. The chemical shifts are given in ppm relative to DMSO d_6_ (^1^H, 3.33 and 2.50 ppm and ^13^C 39.52 ppm). Coupling constants *J* are given in Hz. Multiplicities are given as follows: singlet (s), doublet (d), triplet (t), quadruplet (q) and multiplet (m). “CPh” designates protons and carbons of the aromatic ring bound to the carbon atom of imine, and “NPh” designates protons and carbons of the aromatic ring bound to the nitrogen atom of imine (Figure 7).

*General procedure for the synthesis of (hydroxyphenyliminomethyl)phenols***1a**–**1g**.

4-aminophenol (**2)** (1 g, 9.17 mmol) was stirred with an equimolar amount of an aromatic aldehyde **3a***–***g** in 20 mL of distillated water, during 4 to 5 h at room temperature (20 °C). The solid product obtained **1a***–***1g** was filtered, washed with water, air dried and recrystallized from ethanol, acetone, ethyl acetate or acetonitrile.

The ^1^H and ^13^C NMR spectra and the HMBC spectra of aza-stilbenes **1a** to **1g** are shown in Supplementary Materials Figures S1 and S2, respectively.

*4-{[(4-hydroxyphenyl)imino]methyl}phenol* (**1a**): recrystallized from acetone; yield 63%; m.p. 212–214 °C. IR: 3485.4 (ν/O-H), 1639.3 (ν/C = N), 1239.5 (ν/C-O). ^1^H NMR (DMSO-*d*_6_) δ: 10.00 (s, 1H, CPh OH), 9.38 (s, 1H, NPh OH), 8.43 (s, 1H, CH = N), 7.72 (d, 2H, *J* = 8.61 Hz, CPh 2,6-H), 7.11 (d, 2H, *J* = 8.72 Hz, NPh 2,6-H), 6.85 (d, 2H, *J* = 8.58 Hz, CPh 3,5-H), 6.77 (d, 2H, *J* = 8.72 Hz, NPh 3,5-H). ^13^C NMR (DMSO-*d*_6_) δ: 160.1 (CPh C-1), 156.9 (C = N), 155.7 (NPh C-1), 143.2 (NPh C-4), 130.1 (CPh C-2,6), 127.9 (CPh C-4), 122.1 (NPh C-2,6), 115.6 (NPh C-3,5), 115.5 (CPh C-3,5). HRMS (ESI+) *m*/*z*: 214.0861 [M + H]+ calc. for C_13_H_12_NO_2_^+^, 214.0863, found 214.0861.

*3-{[(4-Hydroxyphenyl)imino]methyl}phenol* (**1b**): no recrystallization; yield 87%; m.p. 193–195 °C. IR: 3296.5 (ν/O-H), 1622.2 (ν/C = N), 1214.7 (ν/C-O). 1H NMR (DMSO-d6) δ: 9.61 (s, 1H, CPh OH), 9.47 (s, 1H, NPh OH), 8.50 (s, 1H, CH = N), 7.32 (m, 1H, CPh 4-H), 7.28 (s, 1H, CPh 6-H), 7.27 (m, 1H, CPh 2-H), 7.17 (d, 2H, J = 8.70 Hz, NPh 2,6-H), 6.90–6.86 (m, 1H, CPh 5-H), 6.79 (d, 2H, J = 8.70 Hz, NPh 3,5-H). 13C NMR (DMSO-d6) δ: 157.6 (CPh C-1), 157.2 (C = N), 156.2 (NPh C-1), 142.6 (NPh C-4), 137.8 (CPh C-3), 129.7 (CPh C-6), 122.5 (NPh C-2,6), 119.9 (CPh C-2), 118.1 (CPh C-5), 115.7 (NPh C-3,5), 113.9 (CPh C-4). HRMS (ESI+) *m*/*z*: 214.0862 [M + H]+ calc. for C13H12NO2+, 214.0863, found 214.0862.

*2-{[(4-Hydroxyphenyl)imino]methyl}phenol* (**1c**): no recrystallization; yield 11%; m.p. 138–139 °C. IR: 3392.1 (ν/O-H), 3280.5 (ν/O-H), 1613.8 (ν/C = N), 1209.1 (ν/C-O). 1H NMR (DMSO-d6) δ: 13.41 (s, 1H, CPh OH), 9.67 (s, 1H, NPh OH), 8.90 (s, 1H, CH = N), 7.59 (dd, 1H, J = 7.67, 1.62 Hz, CPh 6-H), 7.37 (m, 1H, CPh 5-H), 7.32 (d, 2H, J = 8.77 Hz, NPh 2,6-H), 6.96 (d, 1H, J = 7.40 Hz, CPh 3-H), 6.93 (d, 1H, J = 8.31 Hz, CPh 4-H), 6.84 (d, 2H, J = 8.75 Hz, NPh 3,5-H). 13C NMR (DMSO-d6) δ: 160.7 (C = N), 160.6 (CPh C-1), 157.4 (NPh C-1), 139.7 (NPh C-4), 133.0 (CPh C-5), 132.7 (CPh C-6), 123.1 (NPh C-2,6), 119.9 (CPh C-2), 119.4 (CPh C-3), 116.9 (CPh C-4), 116.4 (NPh C-3,5). HRMS (ESI+) *m*/*z*: 214.0861 [M + H]+ calc. for C13H12NO2+, 214.0863, found 214.0 861.

*5-{[(4-Hydroxyphenyl)imino]methyl}benzene-1,3-diol* (**1d**): washed with hot ethyl acetate; yield 60%; m.p. decomposed at 238 °C. IR: 3481.2 (ν/O-H), 3273.2 (ν/O-H), 1624.0 (ν/C = N), 1213.8 (ν/C-O). 1H NMR (DMSO-d6) δ: 9.46 (s, 1H, NPh OH), 9.44 (s, 2H, CPh OH), 8.39 (s, 1H, CH = N), 7.16 (d, 2H, J = 8.71 Hz, NPh 2,6-H), 6.78 (d, 2H, J = 8.71 Hz, NPh 3,5-H), 6.76 (d, 2H, J = 2.20 Hz, CPh 2,6-H), 6.32 (t, 1H, J = 2.21 Hz, CPh 4-H). 13C NMR (DMSO-d6) δ: 158.6 (C = N), 157.4 (CPh C-1), 156.1 (NPh C-1), 142.6 (NPh C-4), 138.3 (CPh C-3,5), 122.5 (NPh C-2,6), 115.7 (NPh C-3,5), 106.4 (CPh C-2,6), 105.2 (CPh C-4). HRMS (ESI+) *m*/*z*: 230.0811 [M + H]+ calc. for C13H12NO3+, 230.0812, found 230.0811.

*4-{[(4-Hydroxyphenyl)imino]methyl}anisole* (**1e**): recrystallized from ethanol; yield 40%; m.p. 188–190 °C. IR: 1602.8 (ν/C = N), 1221.3 (ν/C-O). 1H NMR (DMSO-d6) δ: 9.42 (s, 1H, OH), 8.51 (s, 1H, CH = N), 7.83 (d, 2H, J = 8.75 Hz, CPh 2,6-H), 7.15 (d, 2H, J = 8.67 Hz, NPh 2,6-H), 7.04 (d, 2H, J = 8.70 Hz, CPh 3,5-H), 6.78 (d, 2H, J = 8.67 Hz, NPh 3,5-H), 3.82 (s, 3H, CH3). 13C NMR (DMSO-d6) δ: 161.5 (CPh C-1), 156.6 (C = N), 155.9 (NPh C-1), 143.0 (NPh C-4), 130.0 (CPh C-2,6), 129.4 (CPh C-4), 122.3 (NPh C-2,6), 115.7 (NPh C-3,5), 114.2 (CPh C-3,5), 55.4 (CH3). HRMS (ESI+) *m*/*z*: 228.1018 [M + H]+ calc. for C14H14NO2+, 228.1019, found 228.1018.

*4-{[(4-Hydroxyphenyl)imino]methyl}bromobenzene* (**1f**): recrystallized from acetonitrile; yield 33%; m.p. 205–206 °C. IR: 1618.7 (ν/C = N), 1223.9 (ν/C-O). 1H NMR (DMSO-d6) δ: 9.54 (s, 1H, OH), 8.61 (s, 1H, CH = N), 7.84 (d, 2H, J = 8.48 Hz, CPh 2,6-H), 7.70 (d, 2H, J = 8.40 Hz, CPh 3,5-H), 7.22 (d, 2H, J = 8.70 Hz, NPh 2,6-H), 6.80 (d, 2H, J = 8.70 Hz, NPh 3,5-H). 13C NMR (DMSO-d6) δ: 156.5 (CPh C-1), 155.9 (C = N), 142.2 (NPh C-1), 135.7 (NPh C-4), 131.8 (CPh C-3,5), 130.0 (CPh C-2,6), 124.3 (CPh C-4), 122.7 (NPh C-2,6), 115.7 (NPh C-3,5). HRMS (ESI+) *m*/*z*: 276.0018 [M + H]+ calc. for C13H11NOBr+, 276.0019, found 276.0018.

*2-{[(4-Hydroxyphenyl)imino]methyl}bromobenzene* (**1g**): recrystallized from acetonitrile; yield 35%; m.p. 152–153 °C. IR: 1613.7 (ν/C = N), 1236.2 (ν/C-O). 1H NMR (500 MHz, DMSO-d6) δ: 9.62 (s, 1H, OH), 8.79 (s, 1H, CH = N), 8.11 (dd, 1H, J = 7.75 Hz, CPh 6-H), 7.74 (dd, 1H, J = 7.94 Hz, CPh 63H), 7.50 (t, 1H, J = 7.43 Hz, CPh 5-H), 7.44 (td, 1H, J = 7.61 Hz, CPh 4-H), 7.23 (d, 2H, J = 8.73 Hz, NPh 2,6-H), 6.83 (d, 2H, J = 8.73 Hz, NPh 3,5-H). 13C NMR (126 MHz, DMSO-d6) δ: 156.9 (CPh C-1), 155.0 (C = N), 142.2 (NPh C-1), 134.3 (CPh C-2), 133.2 (CPh C-3), 132.6 (CPh C-4), 128.5 (CPh C-6), 128.1 (CPh C-5), 125.0 (NPh C-4), 122.7 (NPh C-2,6), 115.9 (NPh C-3,5). HRMS (ESI+) *m*/*z*: 276.0018 [M + H]+ calc. for C13H11NOBr+, 276.0019, found 276.0018.

### 4.2. Cell Culture and Treatments

The mouse neuro-2a (N2a) neuroblastoma cell line (Ref: CCL-131, American Type Culture Collection (ATCC), Manassas, VA, USA) was maintained in Dulbecco’s modified Eagle medium (DMEM, Lonza, Amboise, France) containing 10% (*v/v*) of heat-inactivated fetal bovine serum (FBS) (Pan Biotech, Aidenbach, Germany) (30 min, 56 °C) and 1% (*v/v*) of penicillin (100 U/mL)/streptomycin (100 mg/mL) (Pan Biotech). The cells were incubated at 37 °C in a humidified atmosphere (5% CO_2_, 95% air) and passaged twice a week. The cells were seeded at 60,000 cells per well containing 1 mL of DMEM supplemented with 10% (*v/v*) heat-inactivated FBS and 1% antibiotics (penicillin, streptomycin) in 24-well plates (FALCON, Becton Dickinson, Franklin Lakes, NJ, USA). The stock solutions of resveratrol (trans-resveratrol, **RSV**) and aza-stilbenes (**AZA-ST 1a** to **1g**) were prepared as follows: resveratrol (reference of the product: 501-36-0; purity 99%; Sigma-Aldrich, St Quentin-Fallavier, France) was prepared at 50 mM in absolute ethanol (EtOH; Carlo Erba Reagents, Val de Reuil, France); **AZA-ST 1a** to **1g** were prepared at 50 mM in dimethyl sulfoxide (DMSO; Sigma-Aldrich). The aspects of resveratrol and aza-stilbenes in solution are shown in supplementary Figure S3. In order to evaluate the effects of aza-stilbenes on N2a cells comparatively to resveratrol (effects on cell density, esterase activity and transmembrane mitochondrial potential (ΔΨm)), the growth medium was removed after 24 h of culture and the N2a cells were incubated either with resveratrol or aza-stilbenes used at various concentrations ranging from 1.5 to 100 μM for 48 h. The highest concentration of 100 µM is obtained by diluting 16 µL of stock solution (resveratrol or aza-stilbene; stock solution at 50 mM) in 8 mL of culture medium. Concentrations of 50 to 1.5 μM are obtained by successive dilution in cascade of two by two in culture medium. The effects of vehicles (DMSO, ethanol) were evaluated at their highest concentration (0.2% *v/v*).

### 4.3. Measurement of Cell Density: Sulforhodamine 101 (SR101) Assay

Sulforhodamine 101 (SR101) (reference of the product: S7635, Sigma Aldrich) assay was used to assess the cytotoxic effect of resveratrol and AZA-ST on N2a cells at different concentrations ranging from 1.5 to 100 µM. SR101 is an anionic dye that electrostatically binds to cellular proteins [35,36]. SR101 permits the quantification of adherent cells, considered as viable cells, since cell death is associated with a loss of cell adhesion. The experiments were realized four times in triplicate. The data were expressed as percentage of the control.

### 4.4. Measurement of Esterase Activity: Fluorescein Diacetate (FDA) Assay

Cell viability was measured with the fluorescein diacetate (FDA) (reference of the product: F1303, Invitrogen/Molecular Probes) assay, which considers esterase [35,41]. The N2a cells, previously cultured for 24 h in 24-well plates in DMEM containing 10% FBS, were further incubated for 48 h, with and without resveratrol, or AZA-ST, used at different concentrations (1.5 to 100 μM). At the end of treatment, cells were incubated in the dark with 15 µg/mL FDA for 5 min at 37 °C, rinsed twice with phosphate buffered saline (PBS), then lysed with 10 mM Tris-HCl solution containing 1% sodium dodecyl containing 1% sodium dodecyl sulfate (SDS) for 10 min. Using a TECAN fluorescence microplate reader (Sunrise spectrophotometer, TECAN, Lyon, France), the fluorescence intensity was measured with an excitation at 485 nm and an emission at 528 nm. All assays were performed in three independent experiments and performed in triplicate. Data were expressed as percentage of untreated cells (control).

### 4.5. Measurement of Transmembrane Mitochondrial Potential (ΔΨm): 3,3′-Dihexyloxacarbocyanine IODIDE (DiOC_6_(3)) Assay

The variation of the mitochondrial transmembrane potential (∆Ψm) was measured using 3,3′-dihexyloxacarbocyanine iodide (DiOC_6_(3)) (D273, Invitrogen/Thermo Fisher Scientific, Montigny le Bretonneux, France). This fluorochrome accumulates in the mitochondria proportionally to the ΔΨm value [42]. The higher the ∆Ψm, the more the probe accumulates. After 48 h of treatment, adherent cells collected by trypsinization were pooled with non-adherent cells and stained with a solution of DiOC_6_(3) at 40 nM (15 min; 37 °C). The cells were immediately analyzed on a BD Accuri™ C6 flow cytometer (BD Biosciences, San Jose, CA, USA). The loss of ∆Ψm is indicated by a decrease in the intensity of the green fluorescence collected through a band pass filter of 520 ± 10 nm. For each sample, 10,000 cells were acquired, and the data were analyzed with FlowJo (Tree Star Inc., Carrboro, NC, USA) software. All assays were performed in triplicate.

### 4.6. Measurement of Antioxidant Activity with the KRL (Kit Radicaux Libres) Assay

The KRL (Kit Radicaux Libres) test was used to assess the oils’ overall antioxidant activity by their ability to protect erythrocytes against a controlled free radical attack at varying concentrations [20,21]. Diluted control blood samples were exposed to organic free radicals generated at 37 °C from the thermal decomposition of a solution of 2.2′–azobis (2-amidinopropane) dihydrochloride (AAPH). In order to record haemolysis, the turbidimetric optical density decline at 620 nm was measured using a 96-well microplate reader (KRL Reader, Kirial International; Couternon, France). The antioxidant activity of the tested oil samples was expressed in Trolox equivalents and gallic acid equivalent. Lara-Spiral laboratory performed the KRL test (Couternon, France).

### 4.7. Measurement of Antioxidant Activity with the DPPH (2,2′-Diphenyl-1-Picrylhydrazyl Radical) Assay

The DPPH• radical is a stable soluble molecule characterized by its deep-violet color, with an absorption maximum at 515 nm. Antioxidants (AH) or other radical species (R•) are able to react with this stable radical (DPPH•) by providing an electron or hydrogen atom, thus reducing it to 2,2-diphenyl-1-hydrazine (DPPH-H) or a substituted analogous hydrazine (DPPH-R) characterized by colorless or pale-yellow color that could be easily monitored with a spectrophotometer. In the present study, the free radical scavenging activity was determined by the DPPH assay as previously described [22]. DPPH• was prepared in 95% methanol and protected from light. In 96-well plates, 40 µL of DPPH (0.2 mmol/L) was added to 160 µL of sample or blank, and the mixture was homogenized and left to stand in the dark for 30 min. Absorbance was measured using a spectrophotometer at 517 nm, and DPPH• radical scavenging activity was expressed as a percent of inhibition (PI) using the following equation:

PI = [(A0 − A1)/A0] × 100

where A0 is the absorbance of the DPPH solution, and A1 is the absorbance of the DPPH solution after the addition of the sample. All assays were performed in three independent experiments and performed in triplicate.

### 4.8. Measurement of Antioxidant Activity with the Ferric Reducing Antioxidant (FRAP) Assay

Ferric reducing antioxidant power (FRAP) is based on the reduction of Fe^3+^ (ferric ions) to Fe^2+^ (ferrous ions), and the assay was carried using previously described methods [23,24]. At low pH, in the presence of 2,4,6-Tris(2-pyridyl)-s-triazine (TPTZ, Sigma Aldrich, France), the ferric-tripyridyltriazine complex (Fe^3+^-TPTZ) is reduced to ferrous (Fe^2+^-TPTZ) with the formation of an intense blue color with an absorption maximum at 593 nm. Briefly, 2.3 mL of FRAP reagent (contains 100 mL of 0.3 M acetate buffer, pH 3.6; 20 mL of 10 mM TPTZ solution in 40 mM HCl; and 20 mL of 20 mM FeCl_3_) was mixed with 0.7 mL of the product (resveratrol or aza-stilbene **1a***–***1g**) at different concentrations. The mixture was then incubated at 37 °C for 30 min in the dark. The absorbance was measured at 593 nm against a blank containing all the reagents, except the sample, using a spectrophotometer (Safas Xenus, Monaco). The increase in absorbance of the reaction mixture indicates an increase in reduction capacity. Results of the samples were expressed as µMol Fe^2+^ reduced per mM of resveratrol and aza-stilbenes from a standard calibration curve of FeSO_4_∙7H_2_O.

### 4.9. Measurement of Antioxidant Activity with the PAOT Liquid^®^ Technology Assay

The total antioxidant power of resveratrol and aza-stibenes was also determined by PAOT Liquid^®^ Technology (patent FR1871986; 11.28.2018; https://worldwide.espacenet.com/patent/search/family/066776410/publication/US2022031230A1?q=FR1871986; accessed on 19 July 2022). This method was based on an electrochemical reaction with a molecule in a free radical state (mediator M•), following Equation 1 (Equation (1)) [25].

Oxidized mediator M• + AOX → Reduced mediator M + oxidized AOX (1)

Analysis was started with electrochemical potential measurement (EP control t0) of the reaction medium (1 mL), and then 20 µL of sample was added. The potential product (EP product t4) was registered after 4 min of interaction between the sample that contains antioxidants and reduced mediator M•

The variation ratio of the oxidized and reduced forms of the mediator M• during reaction gives an estimation of antioxidant activity in the sample by using Equation 2 (Equation (2))

Antioxidant activity = X 100% (2)

The results were expressed as PAOT score per liter (PAOT Score/L) of the analyzed sample.

### 4.10. Statistical Analysis

The experimental results were statistically analyzed with GraphPad Prism 8.0 software (GraphPad Software, San Diego, CA, USA). Data were expressed as the mean ± standard deviation (SD) and compared with a Student’s t-test. A *p*-value less than 0.05 was considered statistically significant.

## Figures and Tables

**Table 1:** IC50 values of resveratrol and aza-stilbenes **1a** to **1g** obtained with the SR101, FDA et DiOC_6_(3) assays. Sulforhodamine 101 (SR101, evaluation of cell density), fluorescein diacetate (FDA, esterase activity/evaluation of cell viability) and DiOC_6_(3) (measurement of transmembrane mitochondrial potential (ΔΨm)). Aza-stilbenes were compared with resveratrol (*trans-*resveratrol).

	Molecular Formula	Molecular Weight (g/mol)	IC50 µM (SR101)	IC50 µM (FDA)	IC50 µM (DiOC_6_(3))
Resveratrol	C_14_H_12_O_3_	228.24	~12.5	6.25 < IC50 < 12.5	100
Aza-Stilbene 1a	C_13_H_11_NO_2_	213.228	25 < IC50 < 50	12.5	50 < IC50 < 100
Aza-Stilbene 1b	C_13_H_11_NO_2_	213.228	12.5	12.5 < IC50 < 25	50 < IC50 < 100
Aza-Stilbene 1c	C_13_H_11_NO_2_	213.228	25	12.5 < IC50 < 25	50 < IC50 < 100
Aza-Stilbene 1d	C_13_H_11_NO_3_	229.228	25	6.25	50 < IC50 < 100
Aza-Stilbene 1e	C_13_H_10_NOBr	227.254	25 < IC50 < 50	6.25 < IC50 < 12.5	50 < IC50 < 100
Aza-Stilbene 1f	C_13_H_10_NOBr	276.11	25 < IC50 < 50	12.5	50 < IC50 < 100
Aza-Stilbene1g	C_13_H_10_NOBr	276.11	6.25	~12.5	50 < IC50 < 100

**Table 2:** Antioxidant activities of resveratrol and aza-stilbenes.

	Antioxidant Activities of Resveratrol and Aza-Stilbenes 1a–1g Evaluated with Different Assays			
Compounds	KRL (20 µM)	DPPH (25 µM)	FRAP (25 µM)	PAOT Score (25 µM)
Resveratrol	72.81 ± 1.10	32.76 ± 6.40	10.41 ± 0.05	19.39 ± 1.06
Aza-stilbene 1a	129.74 ± 3.92	35.07 ± 1.75	10.52 ± 0.05	42.65 ± 0.78
Aza-stilbene 1b	141.29 ± 4.65	38.84 ± 1.90	10.02 ± 0.04	34.11 ± 0.18
Aza-stilbene 1c	128.38 ± 2.03	5.51 ± 2.25	12.32 ± 0.06	13.34 ± 0.54
Aza-stilbene 1d	154.63 ± 3.50	47.84 ± 2.35	11.05 ± 0.07	56.49 ± 0.59
Aza-stilbene 1e	116.06 ± 4.40	48.55 ± 2.40	10.34 ± 0.05	ND
Aza-stilbene 1f	123.78 ± 5.16	53.69 ± 2.65	10.41 ± 0.05	ND
Aza-stilbene 1g	125.76 ± 3.39	17.23 ± 0.85	10.18 ± 0.04	ND

Aza-stilbenes and resveratrol were used either at 20 or 25 µM. No differences were observed between resveratrol and aza-stilbenes with the FRAP assays. According to the KRL assay, DPPH assay and PAOT, various antioxidant properties of aza-stilbenes and resveratrol were identified. The KRL assay is expressed as increase of haemolysis time (% of control); DPPH assay: % inhibition; FRAP assay: µMol Fe^2+^ reduced per mM of resveratrol and aza-stilbenes; PAOT score: (PAOT Score/L) of analyzed sample. Data are means of 1–2 independent experiments realized in triplicate. N.D: not determined.

## Data Availability

All data are available at the Institute of Molecular Chemistry of University of Burgundy (ICMUB UMR CNRS 6302; Dijon, France) and at Laboratory Bio-peroxIL (University of Burgundy/Inserm, Dijon, France).

## Supplementary Materials

The following supporting information can be downloaded at: https://www.mdpi.com/article/10.3390/molecules27154713/s1, 1H (500 MHz), 13C (126 MHz) and HMBC NMR spectra of the seven aza-stilbenes synthethized; aspects of resveratrol and aza-stilbenes in solution.

## Sample Availability

Samples of the compounds **1a**–**1g** are available from the authors.

## References

[1] Quideau S., Deffieux D., Douat-Casassus C., Pouységu L. (2011). Plant polyphenols: Chemical properties, biological activities and synthesis. Angew. Chem..

[2] Lucarini M., Durazzo A., Lombardi-Boccia G., Souto E.B., Cecchini F., Santini A. (2021). Wine plolyphenols and health: Quantitative research literature analysis. Appl. Sci..

[3] Gao Y., Yu X., Wang B., Yin G., Wang J., Wang T., Bi K. (2021). Based on multi-activity integrated strategy to screening, characterization and quantification of bioactive compounds from red wine. Molecules.

[4] Colica C., Milanovic M., Milié N., Aiello V., De Lorenzo A., Abenavoli L. (2018). A systematic review on natural antioxidant properties of resveratrol. Nat. Prod. Commun..

[5] Yamine A., Namsi A., Vervandier-Fasseur D., Mackrill J.J., Lizard G., Latruffe N. (2021). Polyphenols of the mediterranean diet and their metabolites in the prevention of colorectal cancer. Molecules.

[6] Pezzuto J.M. (2019). Resveratrol: Twenty years of growth, development and controversy. Biomol. Ther..

[7] Walle T. (2011). Bioavailability of resveratrol. Ann. N.Y. Acad. Sci..

[8] Cardile V., Chillemi R., Lombardo L., Sciuto S., Spatafora C., Tringali C. (2007). Antiproliferative activity of methylated analogues of *E-* and *Z*-resveratrol. Z. Naturforsch. C.

[9] Liu Q., Kim C.T., Jo Y.H., Kim S.B., Hwang B.Y., Lee M.K. (2015). Synthesis and biological evaluation of resveratrol derivatives as melanogenesis inhibitors. Molecules.

[10] Nawaz W., Zhou Z., Deng S., Ma X., Ma X., Li C., Shu X. (2017). Therapeutic versatility of resveratrol derivatives. Nutrients.

[11] Chalal M., Vervandier-Fasseur D., Meunier P., Cattey H., Hierso J.C. (2012). Syntheses of polyfunctionalized resveratrol derivatives using Wittig and Heck protocols. Tetrahedron.

[12] Chalal M., Klinguer A., Echairi A., Meunier P., Vervandier-Fasseur D., Adrian M. (2014). Antimicrobial activity of resveratrol analogues. Molecules.

[13] Chalal M., Delmas D., Meunier P., Latruffe N., Vervandier-Fasseur D. (2014). Inhibition of cancer derived cell lines proliferation by newly synthesized hydroxylated stilbenes and ferrocenyl-stilbene analogs. Comparison with resveratrol. Molecules.

[14] Latruffe N., Vervandier-Fasseur D. (2018). Strategic syntheses of vine and wine resveratrol derivatives to explore their effects on cell functions and dysfunctions. Diseases.

[15] Belluti F., Fontana G., Dal Bo L., Carenini N., Giommarelli C., Zunino F. (2010). Design, synthesis and anticancer activities of stilbene-coumarin hybrid compounds: Identification of novel proapopoptic agents. Bioorg. Med. Chem..

[16] Bellina F., Guazzelli N., Lessi M., Manzini C. (2015). Imidazole analogues of resveratrol: Synthesis and cancer cell growth evaluation. Tetrahedron.

[17] Li C., Xu X., Wang X.J., Pan Y. (2014). Imine resveratrol analogues: Molecular design, Nrf2 activation and SAR analysis. PLoS ONE.

[18] Bae S.J., Ha Y.M., Kim J.A., Park J.Y., Ha T.K., Park D., Chun P., Park N.H., Moon H.R., Chung H.Y. (2013). A novel synthesized tyrosinase inhibitor: (*E*)-2-((2,4-dihydrophenyl)diazinyl)phenyl-4-methylbenzenesulfonate as an azo-resveratrol analog. Biosci. Biotechnol. Biochem..

[19] Lizard G., Latruffe N., Vervandier-Fasseur D. (2020). Aza- and Azo-stilbenes: Bio-isosteric analogs of resveratrol. Molecules.

[20] Zhang Y., Zou B., Pan Y., Liang H., Yi X., Wang H. (2012). Antioxidant activities and transition metal ion chelating studies of some hydroxyl Schiff base derivatives. Med. Chem. Res..

[21] Siddiqui A., Dandawate P., Rub R., Padhye S., Aphale S., Moghe A., Jagyasi A., Swamy K.V., Singh B., Chatterjee A. (2013). Novel aza-resveratrol analogs: Synthesis, characterization and anti-cancer activity against breast cancer cell lines. Bioorg. Med. Chem. Lett..

[22] Ronghe A., Chatterjee A., Singh B., Dandawate P., Murphy L., Bhat N.K., Padhye S., Bhat H.K. (2014). Differential regulation of estrogen receptors α and β by 4-(E)-{(4-hydroxyphenylimino)-methylbenzene-1,2-diol}, a novel resveratrol analog. J. Steroid Biochem. Mol. Biol..

[23] Tanaka K., Shiraishi R. (2000). Clean and efficient condensation reactions of aldehydes and amines in a water suspension medium. Green Chem..

[24] Lu J., Li C., Chai Y.F., Yang D.Y., Sun C.R. (2012). The anti-oxidant effect of imine resveratrol analogues. Bioorg. Med. Chem. Lett..

[25] Kotora P., Sersen F., Filo J., Loos D., Gregan J., Gregan F. (2016). The scavenging of DPPH, galvinoxyl and ABTS radicals by imine analogs of resveratrol. Molecules.

[26] Yammine A., Zarrouk A., Nury T., Vejux A., Latruffe N., Vervandier-Fasseur D., Samadi M., Mackrill J.J., Greige-Gerges H., Auezova L. (2020). Prevention by Dietary Polyphenols (Resveratrol, Quercetin, Apigenin) Against 7-Ketocholesterol-Induced Oxiapoptophagy in Neuronal N2a Cells: Potential Interest for the Treatment of Neurodegenerative and Age-Related Diseases. Cells.

[27] Zarrouk A., Martine L., Grégoire S., Nury T., Meddeb W., Camus E., Badreddine A., Durand P., Namsi A., Yammine A. (2019). Profile of Fatty Acids, Tocopherols, Phytosterols and Polyphenols in Mediterranean Oils (Argan Oils, Olive Oils, Milk Thistle Seed Oils and Nigella Seed Oil) and Evaluation of their Antioxidant and Cytoprotective Activities. Curr. Pharm. Des..

[28] Alashi A.M., Taiwo K.A., Oyedele D.J., Adebooye O.C., Aluko R.E. (2019). Polyphenol composition and antioxidant properties of vegetable leaf-fortified bread. J. Food Biochem..

[29] Dudonne S., Vitrac X., Coutière P., Woillez M., Mérillon J.M. (2009). Comparative study of antioxidant properties and total phenolic content of 30 plant extracts of industrial interest using DPPH, ABTS, FRAP, SOD, and ORAC assays. J. Agric Food Chem..

[30] Benzie I.F., Strain J.J. (1999). Ferric reducing/antioxidant power assay: Direct measure of total antioxidant activity of biological fluids and modified version for simultaneous measurement of total antioxidant power and ascorbic acid concentration. Methods Enzymol..

[31] Pincemail J., Kaci M.-M., Kevers C., Tabart J., Elle R.E., Meziane S. (2019). PAOT-Liquid^®^ Technology: An Easy Electrochemical Method for Evaluating Antioxidant Capacity of Wines. Diseases.

[32] Caruso F., Tanski J., Villegas-Estrada A., Rossi M. (2004). Structural basis for antioxidant activity of *trans*-resveratrol: Ab initio calculations and crystal and molecular structure. J. Agric. Food Chem..

[33] Rossi M., Caruso F., Opazo C., Salciccioli J. (2008). Crystal and molecular structure of piceatannol: Scavenging features of resveratrol and piceatannol on hydroxyl and peroxyl radicals docking with transthyretin. J. Agric. Food Chem..

[34] Gaenko A.V., Devarajan A., Gagliardi L., Lindh R., Orlandi G. (2007). Ab initio DFT study of *Z-E* isomerization pathways of *N*-benzylideneaniline. Theor. Chem. Acc..

[35] Namsi A., Nury T., Hamdouni H., Yammine A., Vejux A., Vervandier-Fasseur D., Latruffe N., Masmoudi-Kouki O., Lizard G. (2018). Induction of Neuronal Differentiation of Murine N2a Cells by Two Polyphenols Present in the Mediterranean Diet Mimicking Neurotrophins Activities: Resveratrol and Apigenin. Diseases.

[36] Yammine A., Nury T., Vejux A., Latruffe N., Vervandier-Fasseur D., Samadi M., Greige-Gerges H., Auezova L., Lizard G. (2020). Prevention of 7-Ketocholesterol-Induced Overproduction of Reactive Oxygen Species, Mitochondrial Dysfunction and Cell Death with Major Nutrients (Polyphenols, ω3 and ω9 Unsaturated Fatty Acids) of the Mediterranean Diet on N2a Neuronal Cells. Molecules.

[37] Lakey-Beitia J., Burillo A.M., La Penna G., Hegde M.L., Rao K.S. (2021). Polyphenols as Potential Metal Chelation Compounds Against Alzheimer’s Disease. J. Alzheimers Dis..

[38] Uddin M.S., Mamun A.A., Rahman M.M., Jeandet P., Alexiou A., Behl T., Sarwar M.S., Sobarzo-Sánchez E., Ashraf G.M., Sayed A. (2021). Natural Products for Neurodegeneration: Regulating Neurotrophic Signals. Oxid. Med. Cell Longev..

[39] Kaminski J., Lançon A., Aires V., Limagne E., Tili E., Michaille J.J., Latruffe N. (2012). Resveratrol initiates differentiation of mouse skeletal muscle-derived C2C12 myoblasts. Biochem. Pharmacol..

[40] Cho M.R., Lee S., Song S.K. (2022). A Review of Sarcopenia Pathophysiology, Diagnosis, Treatment and Future Direction. J. Korean Med. Sci..

[41] Namsi A., Nury T., Khan A.S., Leprince J., Vaudry D., Caccia C., Leoni V., Atanasov A.G., Tonon M.C., Masmoudi-Kouki O. (2019). Octadecaneuropeptide (ODN) Induces N2a Cells Differentiation through a PKA/PLC/PKC/MEK/ERK-Dependent Pathway: Incidence on Peroxisome, Mitochondria, and Lipid Profiles. Molecules.

[42] Ragot K., Mackrill J.J., Zarrouk A., Nury T., Aires V., Jacquin A., Athias A., Pais de Barros J.P., Vejux A., Riedinger J.M. (2013). Absence of correlation between oxysterol accumulation in lipid raft microdomains, calcium increase, and apoptosis induction on 158N murine oligodendrocytes. Biochem. Pharmacol..

