# Supplementary material for: Cytotoxic and Antioxidant Activities of Imine Analogs of Trans-Resveratrol towards Murine Neuronal N2a Cells
Source: Molecules. 2022 Jul 23;27(15):4713. doi: 10.3390/molecules27154713 (PMC9332718; doi:10.3390/molecules27154713)
Supplement: Supplementary file 1 [file molecules-27-04713-s001.zip › molecules-1808007-supplementary.pdf]

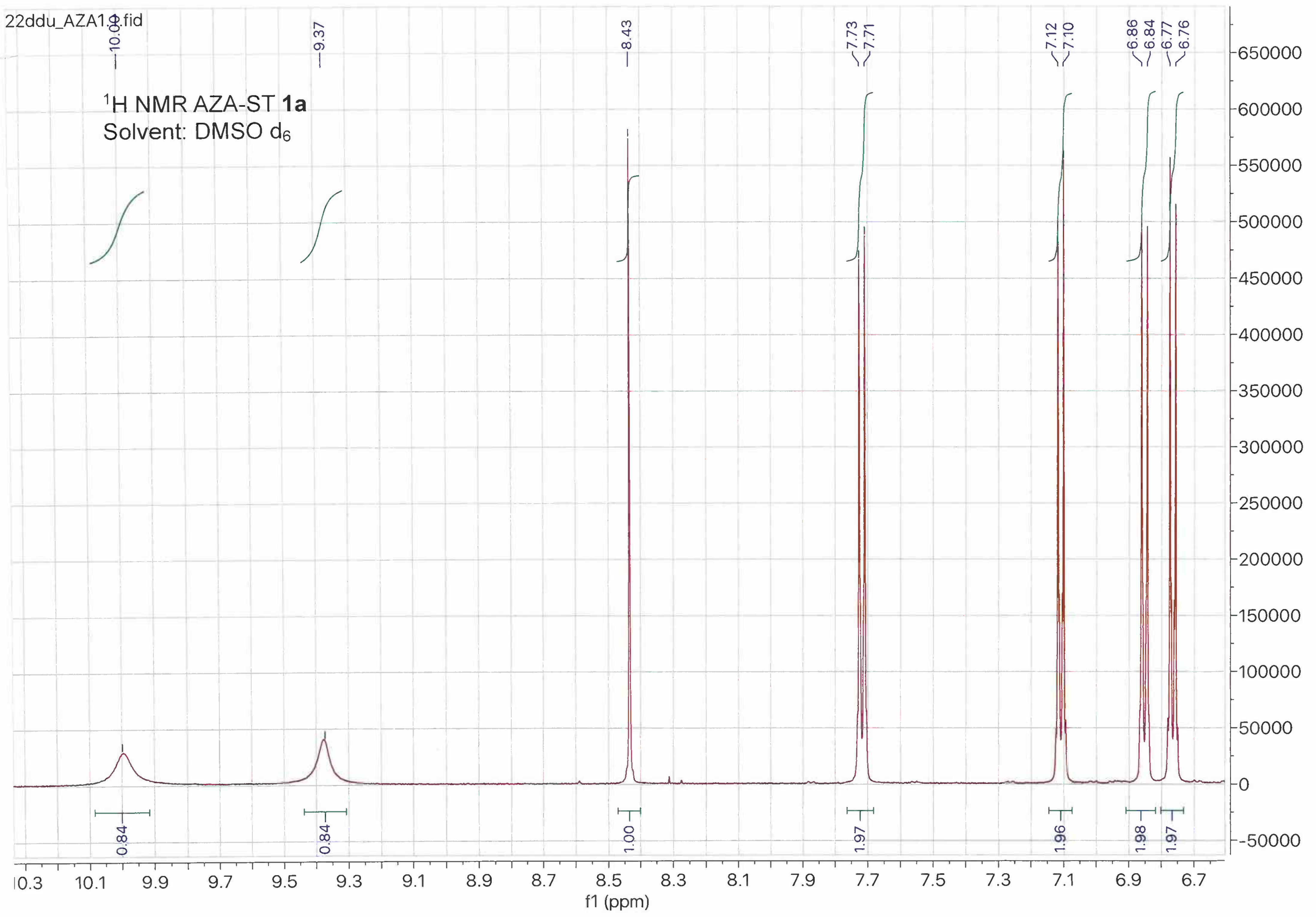

^{1}H NMR AZA-ST **1a**
Solvent: DMSO d_6
9.37
8.43
7.73
7.71
7.12
7.10
6.86
6.84
6.77
6.76
0.84
0.84
1.00
1.97
1.96
1.98
1.97
f1 (ppm)

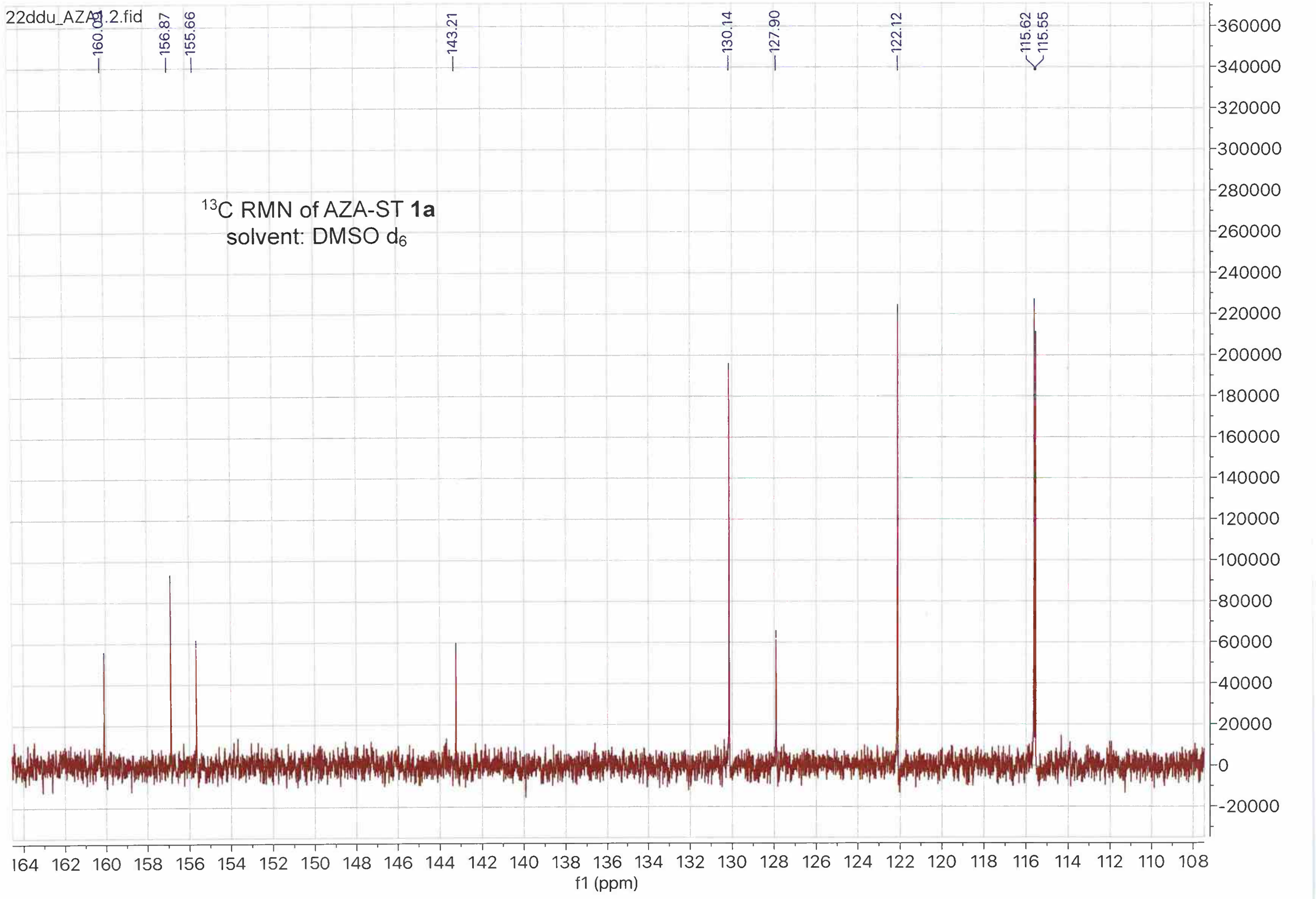
22ddu_AZA[illegible].2.fid
160.0[illegible]
156.87
155.66
143.21
130.14
127.90
122.12
115.62
115.55
^{13}C RMN of AZA-ST **1a**
solvent: DMSO d_6
f1 (ppm)

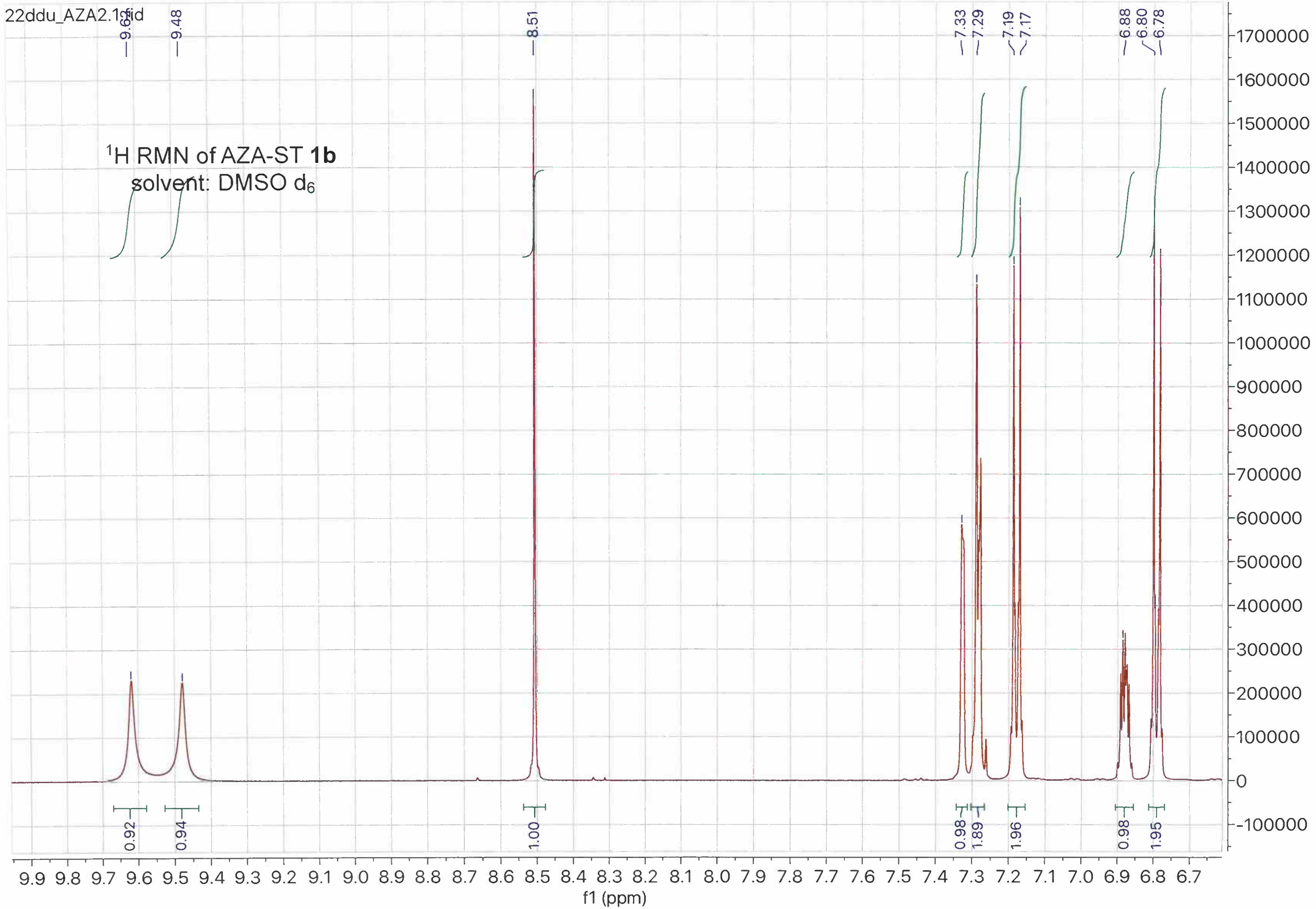

22ddu_AZA2.1fid
9.62
9.48
8.51
7.33
7.29
7.19
7.17
6.88
6.80
6.78
^{1}H RMN of AZA-ST **1b**
solvent: DMSO d6
0.92
0.94
1.00
0.98
1.89
1.96
0.98
1.95
9.9 9.8 9.7 9.6 9.5 9.4 9.3 9.2 9.1 9.0 8.9 8.8 8.7 8.6 8.5 8.4 8.3 8.2 8.1 8.0 7.9 7.8 7.7 7.6 7.5 7.4 7.3 7.2 7.1 7.0 6.9 6.8 6.7
f1 (ppm)
1700000
1600000
1500000
1400000
1300000
1200000
1100000
1000000
900000
800000
700000
600000
500000
400000
300000
200000
100000
0
-100000

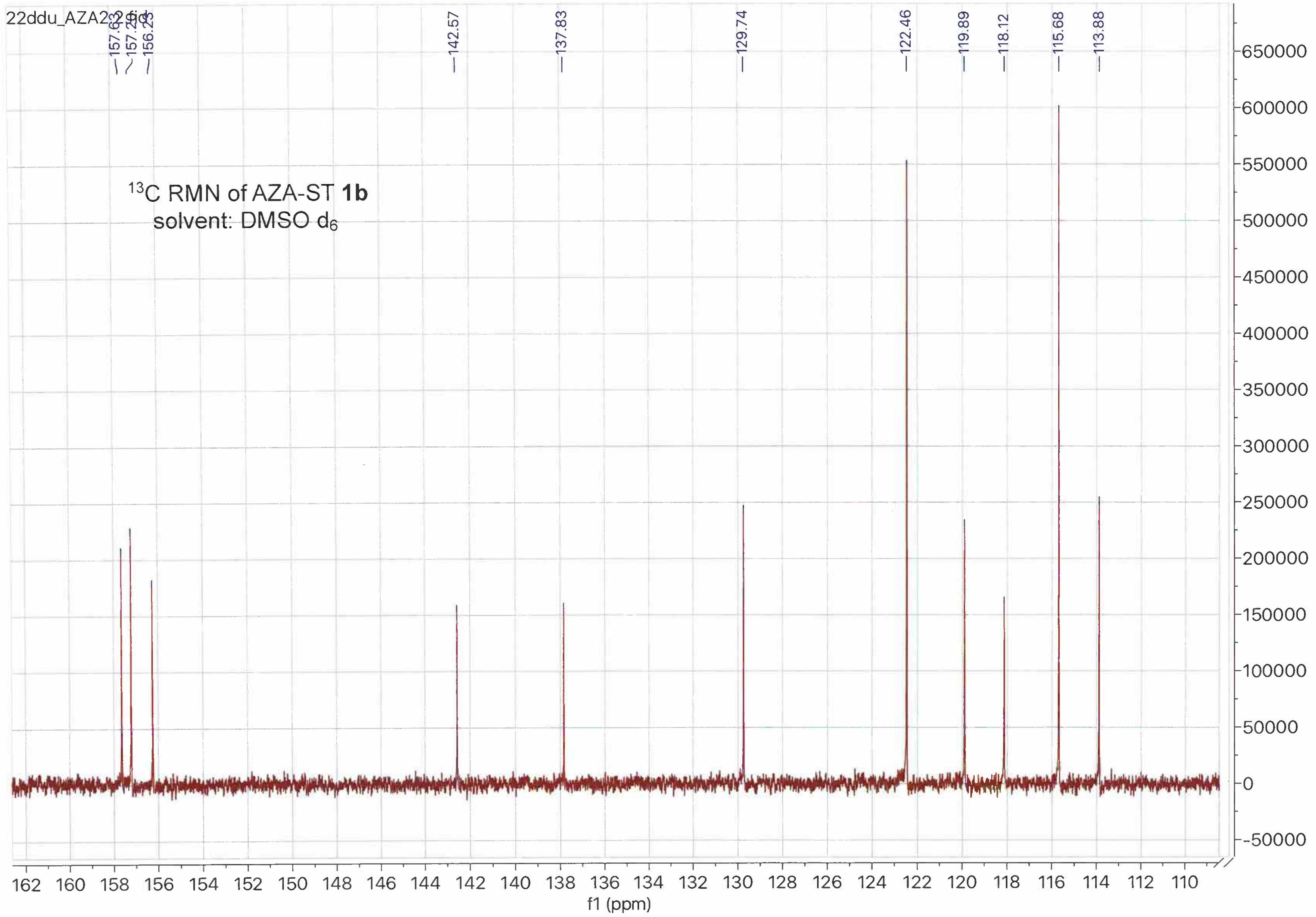
22ddu_AZA22.fid
157.63
157.29
156.23
142.57
137.83
129.74
122.46
119.89
118.12
115.68
113.88
^{13}C RMN of AZA-ST **1b**
solvent: DMSO d_6
650000
600000
550000
500000
450000
400000
350000
300000
250000
200000
150000
100000
50000
0
-50000
162 160 158 156 154 152 150 148 146 144 142 140 138 136 134 132 130 128 126 124 122 120 118 116 114 112 110
f1 (ppm)

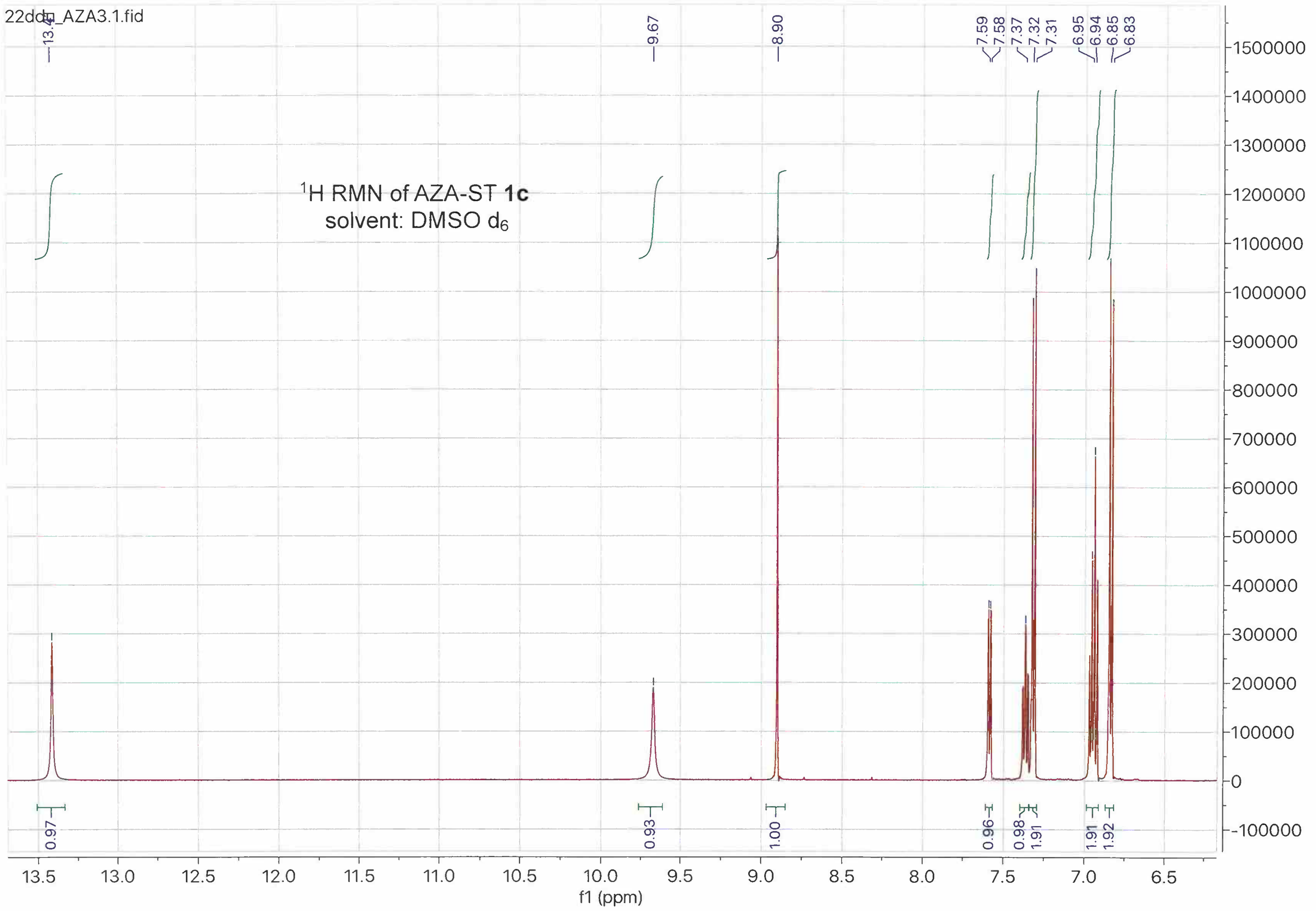
22dd_AZA3.1.fid
13.4
9.67
8.90
7.59
7.58
7.37
7.32
7.31
6.95
6.94
6.85
6.83
1H RMN of AZA-ST 1c
solvent: DMSO d6
0.97
0.93
1.00
0.96
0.98
1.91
1.91
1.92
13.5
13.0
12.5
12.0
11.5
11.0
10.5
10.0
9.5
9.0
8.5
8.0
7.5
7.0
6.5
f1 (ppm)
1500000
1400000
1300000
1200000
1100000
1000000
900000
800000
700000
600000
500000
400000
300000
200000
100000
0
-100000

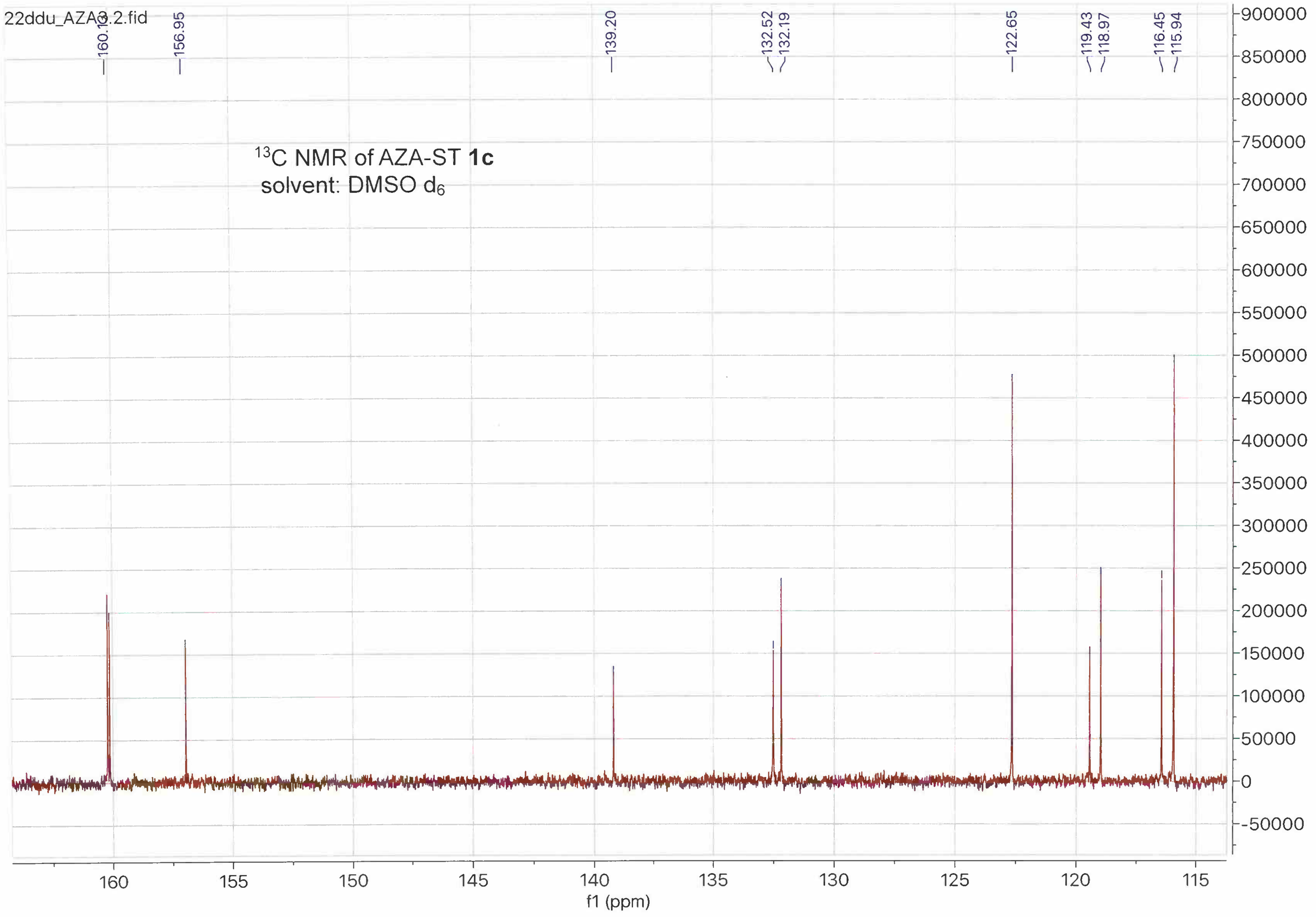
22ddu_AZA3.2.fid
160.1[illegible]
156.95
139.20
132.52
132.19
122.65
119.43
118.97
116.45
115.94
^{13}C NMR of AZA-ST **1c**
solvent: DMSO d_6
900000
850000
800000
750000
700000
650000
600000
550000
500000
450000
400000
350000
300000
250000
200000
150000
100000
50000
0
-50000
160
155
150
145
140
135
130
125
120
115
f1 (ppm)

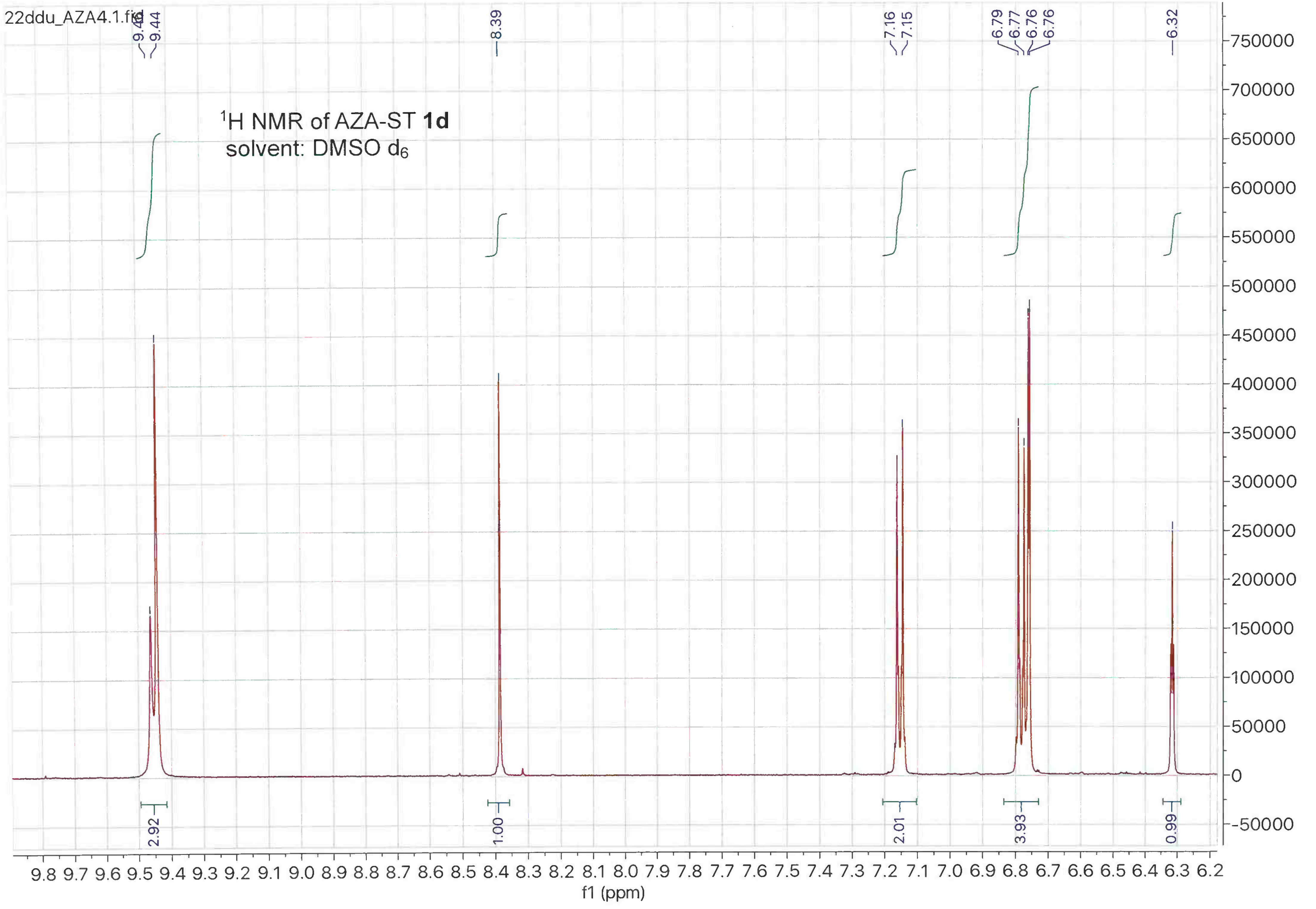

22ddu_AZA4.1.fid
^{1}H NMR of AZA-ST **1d**
solvent: DMSO d_6
9.44
8.39
7.16
7.15
6.79
6.77
6.76
6.76
6.32
2.92
1.00
2.01
3.93
0.99
9.8 9.7 9.6 9.5 9.4 9.3 9.2 9.1 9.0 8.9 8.8 8.7 8.6 8.5 8.4 8.3 8.2 8.1 8.0 7.9 7.8 7.7 7.6 7.5 7.4 7.3 7.2 7.1 7.0 6.9 6.8 6.7 6.6 6.5 6.4 6.3 6.2
f1 (ppm)
750000
700000
650000
600000
550000
500000
450000
400000
350000
300000
250000
200000
150000
100000
50000
0
-50000

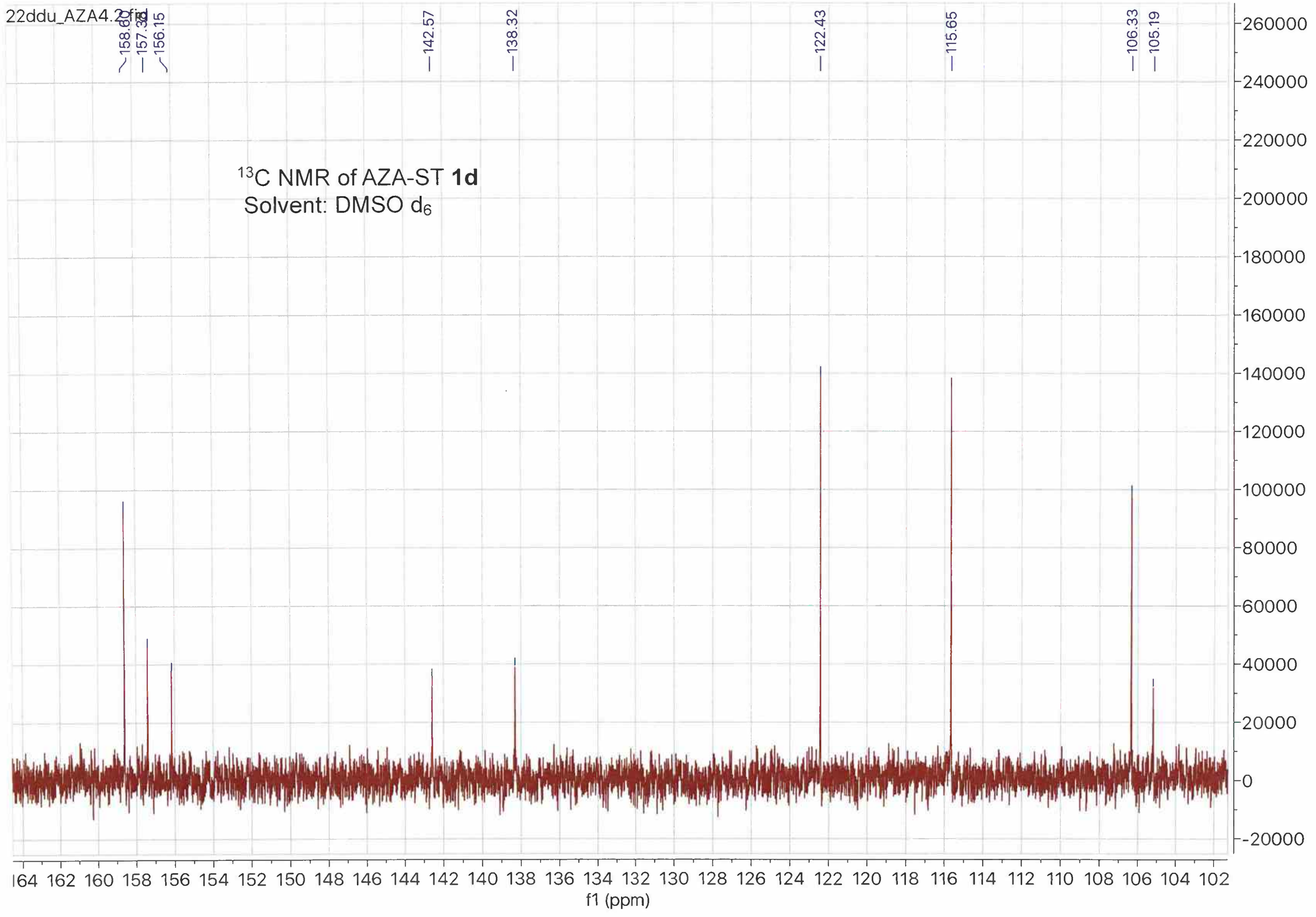
22ddu_AZA4.2.fid
158.60
157.38
156.15
142.57
138.32
122.43
115.65
106.33
105.19
^{13}C NMR of AZA-ST **1d**
Solvent: DMSO d_6
f1 (ppm)
164 162 160 158 156 154 152 150 148 146 144 142 140 138 136 134 132 130 128 126 124 122 120 118 116 114 112 110 108 106 104 102
260000 240000 220000 200000 180000 160000 140000 120000 100000 80000 60000 40000 20000 0 -20000

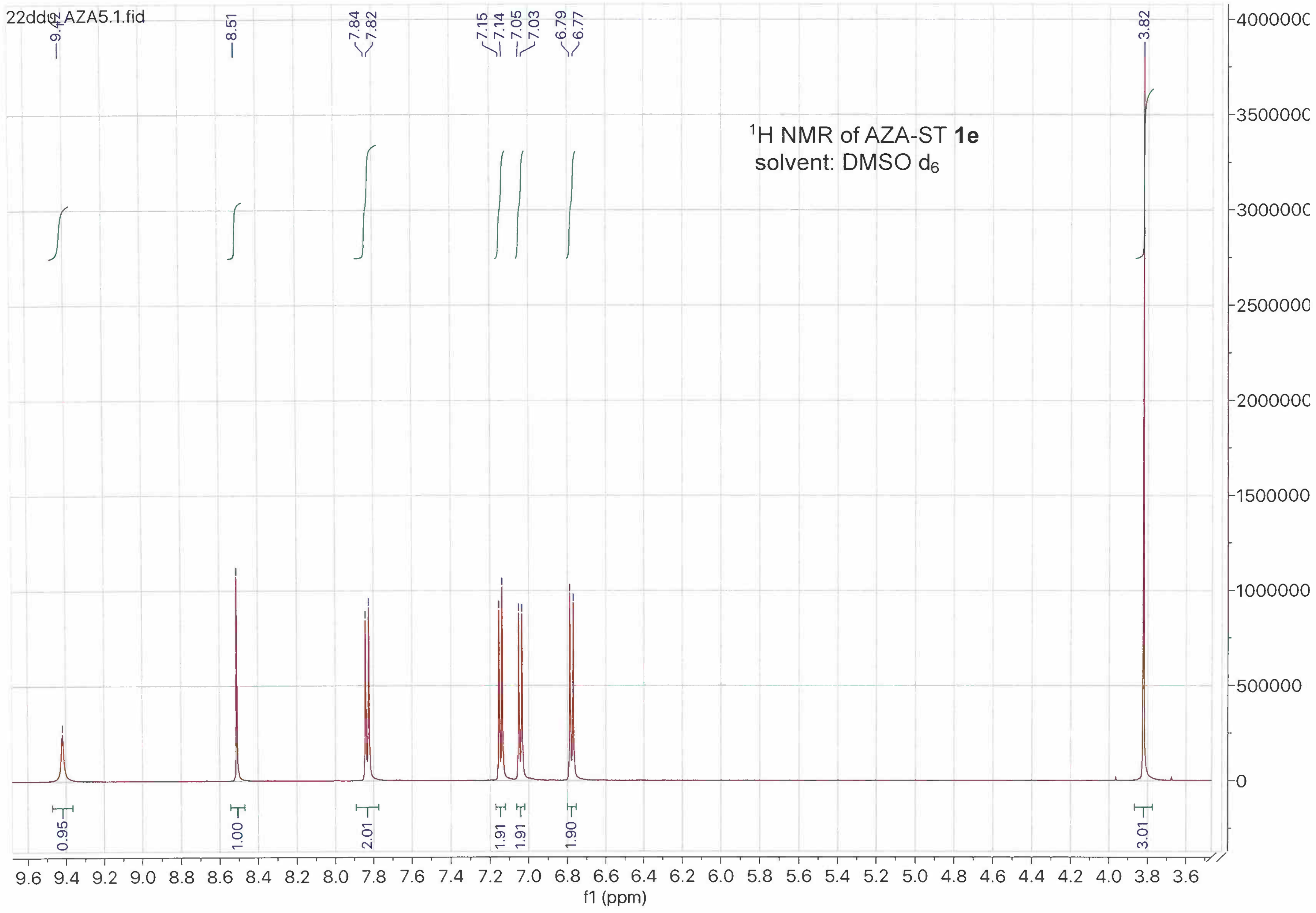
22ddu AZA5.1.fid
9.42
8.51
7.84
7.82
7.15
7.14
7.05
7.03
6.79
6.77
3.82
^{1}H NMR of AZA-ST **1e**
solvent: DMSO d_6
0.95
1.00
2.01
1.91
1.91
1.90
3.01
9.6 9.4 9.2 9.0 8.8 8.6 8.4 8.2 8.0 7.8 7.6 7.4 7.2 7.0 6.8 6.6 6.4 6.2 6.0 5.8 5.6 5.4 5.2 5.0 4.8 4.6 4.4 4.2 4.0 3.8 3.6
f1 (ppm)
4000000
3500000
3000000
2500000
2000000
1500000
1000000
500000
0

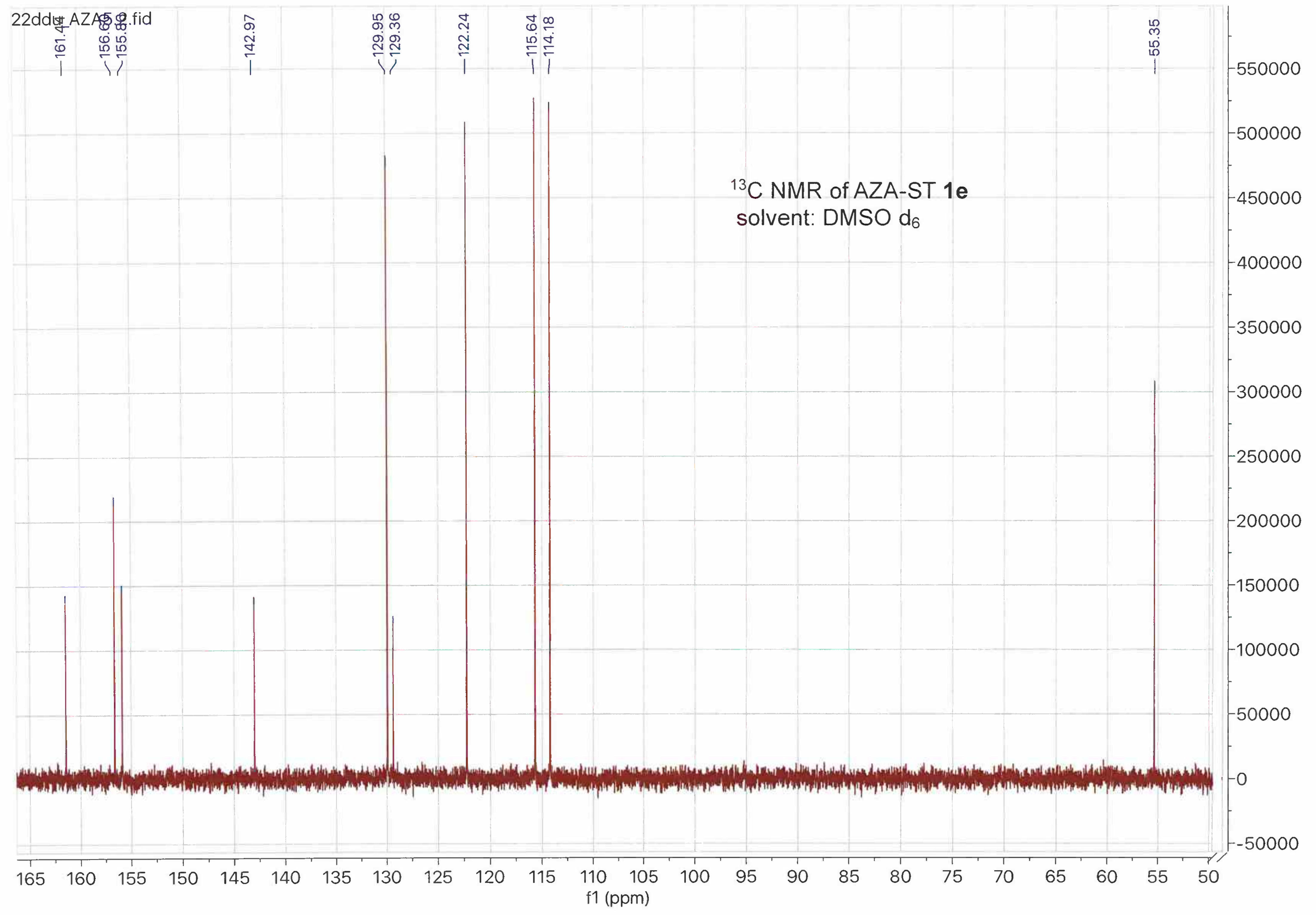

22ddu_AZA5_2.fid
161.44
156.65
155.86
142.97
129.95
129.36
122.24
115.64
114.18
55.35
^{13}C NMR of AZA-ST **1e**
solvent: DMSO d_6
165 160 155 150 145 140 135 130 125 120 115 110 105 100 95 90 85 80 75 70 65 60 55 50
f1 (ppm)
550000 500000 450000 400000 350000 300000 250000 200000 150000 100000 50000 0 -50000

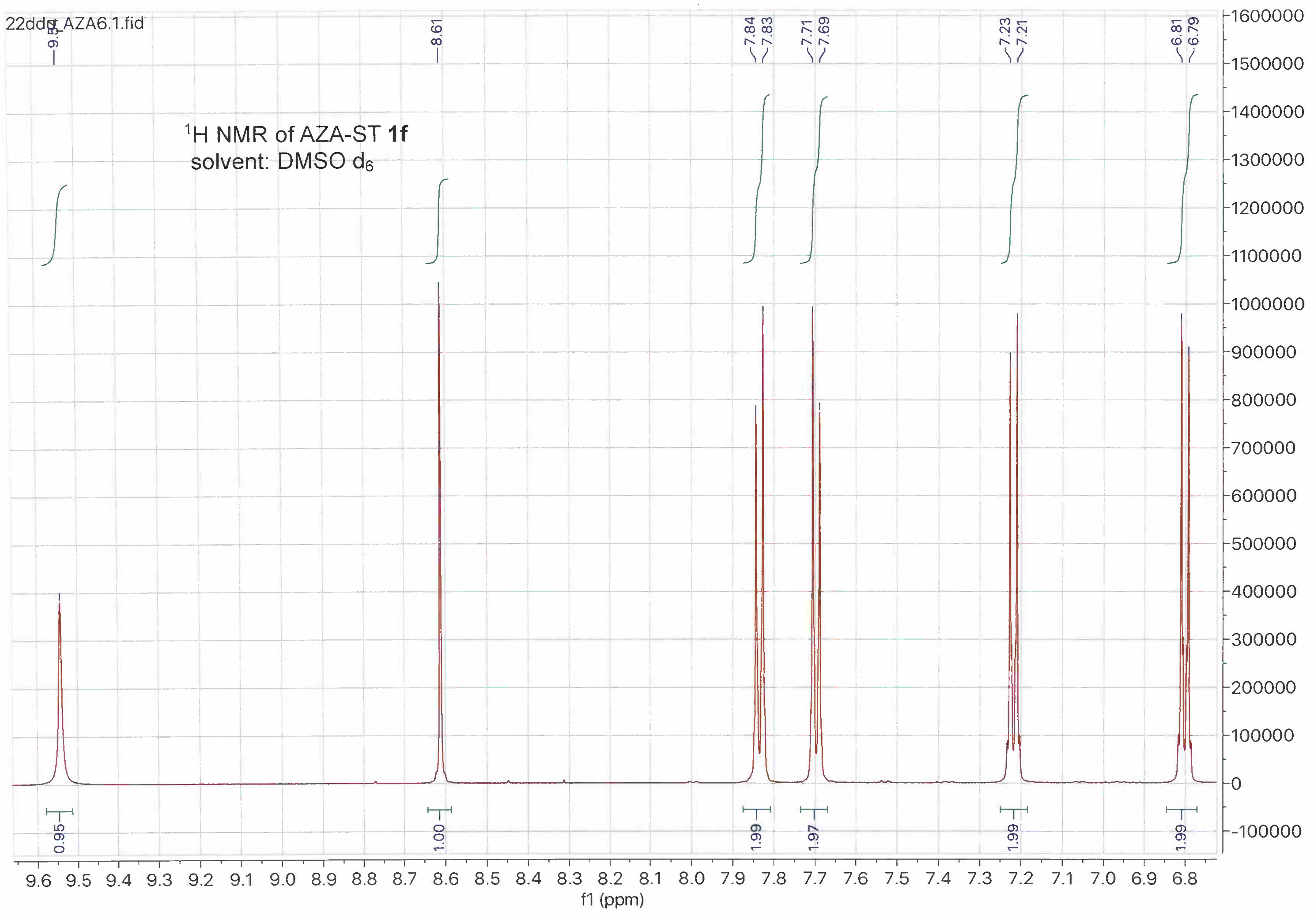

22ddq_AZA6.1.fid
¹H NMR of AZA-ST **1f**
solvent: DMSO d₆
9.55
8.61
7.84
7.83
7.71
7.69
7.23
7.21
6.81
6.79
0.95
1.00
1.99
1.97
1.99
1.99
f1 (ppm)

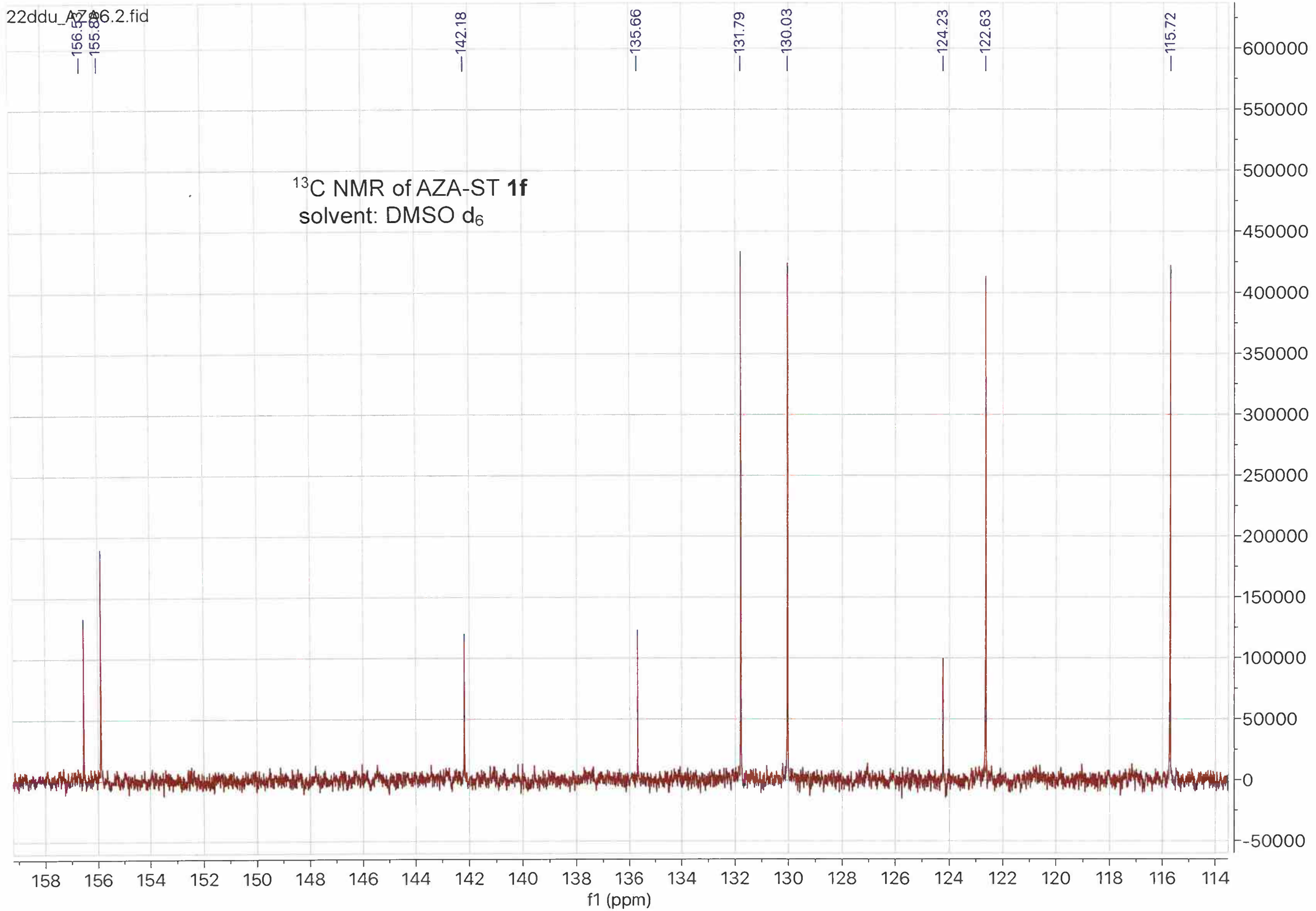
22ddu_AZA6.2.fid
156.5[illegible]
155.8[illegible]
142.18
135.66
131.79
130.03
124.23
122.63
115.72
^{13}C NMR of AZA-ST **1f**
solvent: DMSO d_6
600000
550000
500000
450000
400000
350000
300000
250000
200000
150000
100000
50000
0
-50000
158
156
154
152
150
148
146
144
142
140
138
136
134
132
130
128
126
124
122
120
118
116
114
f1 (ppm)

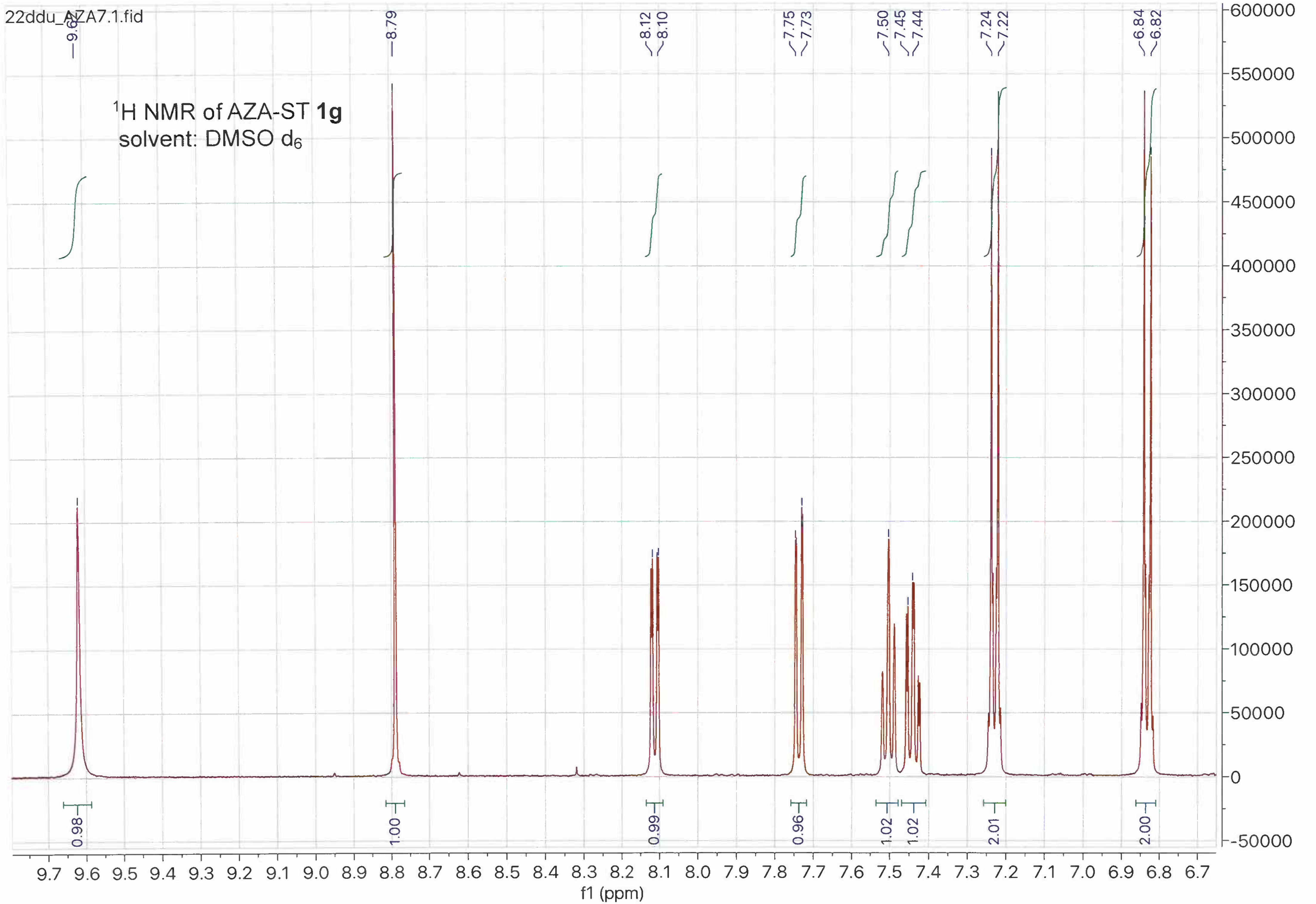
22ddu_AZA7.1.fid
9.62
8.79
8.12
8.10
7.75
7.73
7.50
7.45
7.44
7.24
7.22
6.84
6.82
^{1}H NMR of AZA-ST **1g**
solvent: DMSO d_6
0.98
1.00
0.99
0.96
1.02
1.02
2.01
2.00
9.7 9.6 9.5 9.4 9.3 9.2 9.1 9.0 8.9 8.8 8.7 8.6 8.5 8.4 8.3 8.2 8.1 8.0 7.9 7.8 7.7 7.6 7.5 7.4 7.3 7.2 7.1 7.0 6.9 6.8 6.7
f1 (ppm)
600000
550000
500000
450000
400000
350000
300000
250000
200000
150000
100000
50000
0
-50000

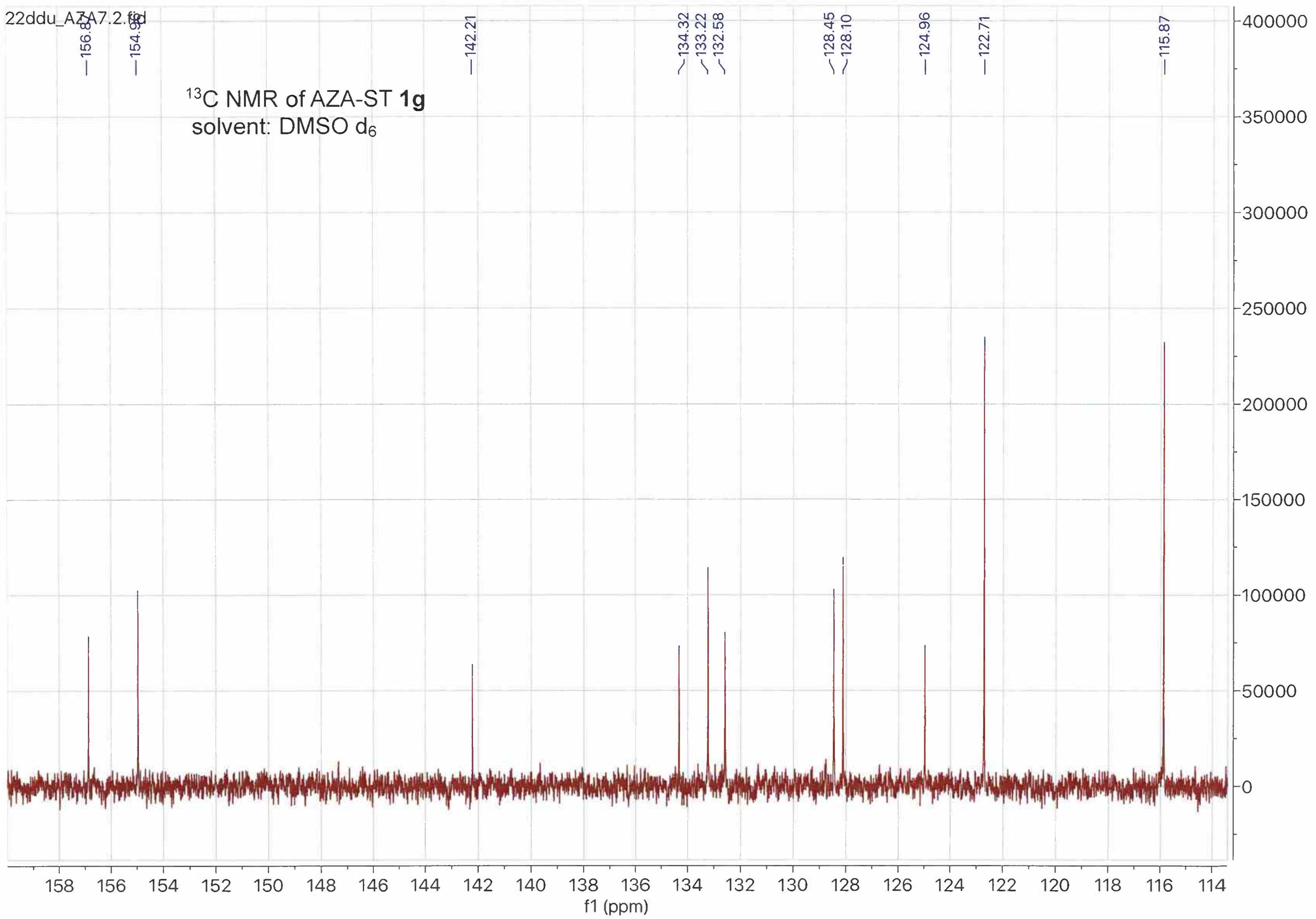

22ddu_AZA7.2.fid
156.87
154.96
142.21
134.32
133.22
132.58
128.45
128.10
124.96
122.71
115.87
^{13}C NMR of AZA-ST **1g**
solvent: DMSO d_6
400000
350000
300000
250000
200000
150000
100000
50000
0
158 156 154 152 150 148 146 144 142 140 138 136 134 132 130 128 126 124 122 120 118 116 114
f1 (ppm)

AZA-ST 1a

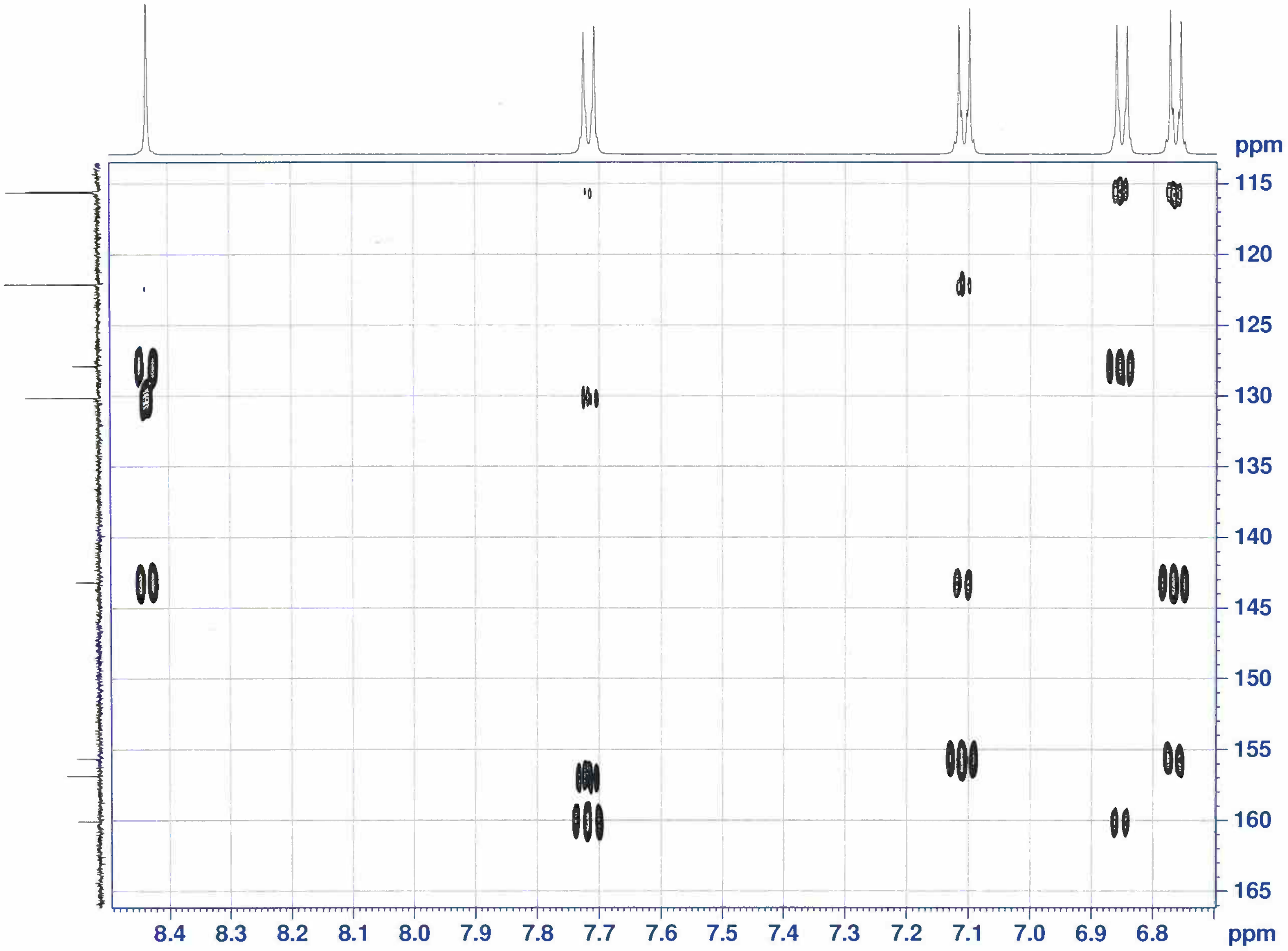


```
Current Data Parameters
NAME          22ddu_AZA1
EXPNO                  7
PROCNO                 1

F2 - Acquisition Parameters
Date_           20220531
Time               21.23 h
INSTRUM        Avance NEO
PROBHD   Z167419_0024 (
PULPROG    hmbcgplpndqf
TD                  4096
SOLVENT             DMSO
NS                    16
DS                    16
SWH             4166.667 Hz
FIDRES          2.034505 Hz
AQ             0.4915200 sec
RG                   101
DW               120.000 usec
DE                  6.50 usec
TE                 297.9 K
CNST2        145.0000000
CNST13        10.0000000
D0            0.00000300 sec
D1            1.50000000 sec
D2            0.00344828 sec
D6            0.05000000 sec
D16           0.00020000 sec
IN0           0.00001809 sec
TDav                   1
SFO1         499.8729707 MHz
NUC1                  1H
P1                  9.73 usec
P2                 19.46 usec
PLW1         32.18000031 W
SFO2         125.7049807 MHz
NUC2                 13C
P3                  9.00 usec
PLW2        106.93000031 W
GPNAM[1]      SMSQ10.100
GPZ1               50.00 %
GPNAM[2]      SMSQ10.100
GPZ2               30.00 %
GPNAM[3]      SMSQ10.100
GPZ3               40.10 %
P16              1000.00 usec

======== F1 INDIRECT DIMENSION ========
td1        128
sw_F1      220.000000

F1 - Acquisition parameters
TD                   128
SFO1             125.705 MHz
FIDRES        432.077423 Hz
SW               219.983 ppm
FnMODE                QF

F2 - Processing parameters
SI                  4096
SF           499.8700018 MHz
WDW                QSINE
SSB                    0
LB                     0 Hz
GB                     0
PC                  1.40

F1 - Processing parameters
SI                  1024
MC2                   QF
SF           125.6924703 MHz
WDW                QSINE
SSB                    0
LB                     0 Hz
GB                     0
```

AZA-ST 1b

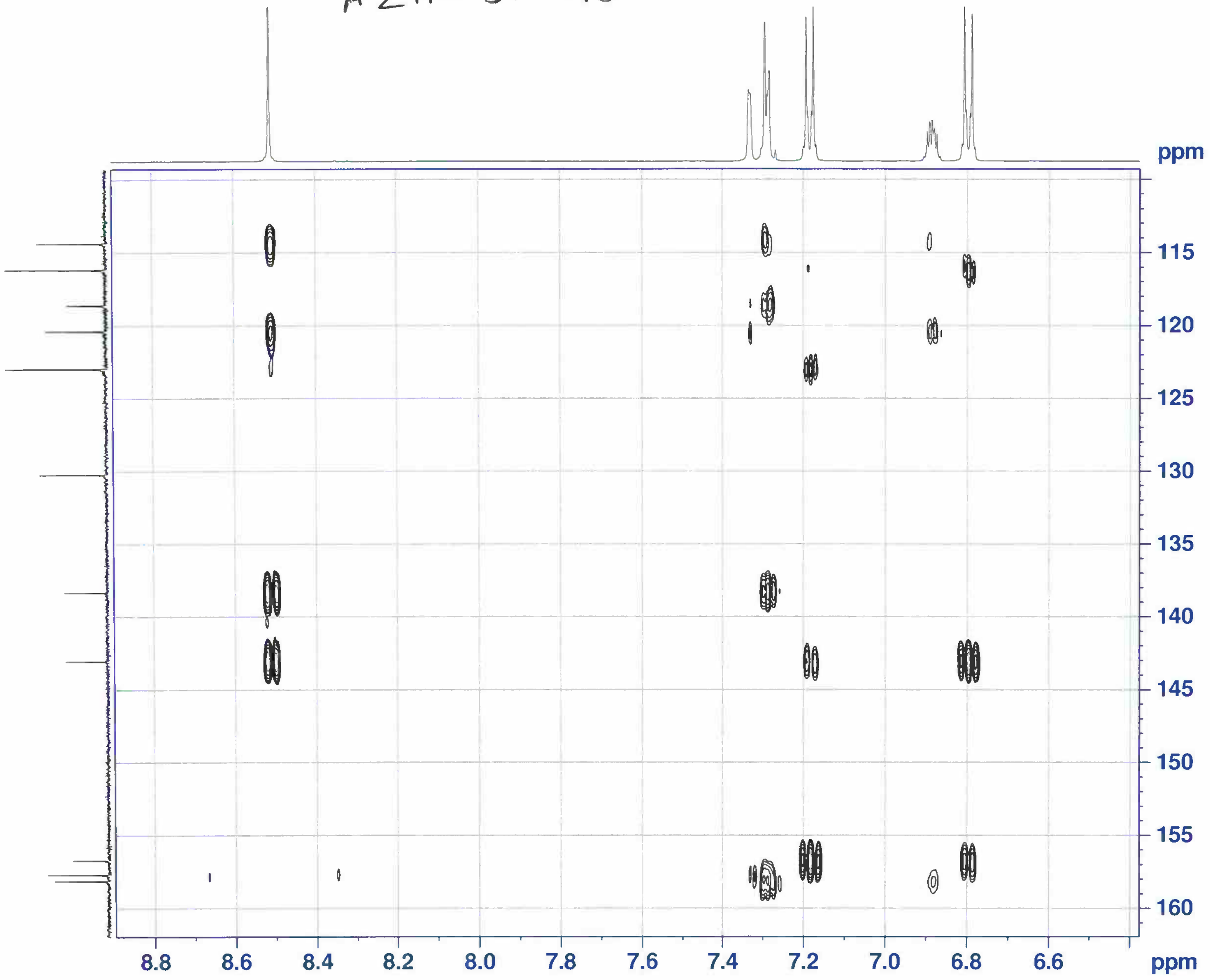


```
Current Data Parameters
NAME             22ddu_AZA2
EXPNO                     7
PROCNO                    1

F2 - Acquisition Parameters
Date_              20220531
Time                  23.41 h
INSTRUM           Avance NEO
PROBHD      Z167419_0024 (
PULPROG        hmbcgplpndqf
TD                     4096
SOLVENT                DMSO
NS                       16
DS                       16
SWH                5263.158 Hz
FIDRES             2.569901 Hz
AQ                0.3891200 sec
RG                      101
DW                   95.000 usec
DE                     6.50 usec
TE                    297.9 K
CNST2           145.0000000
CNST13           10.0000000
D0               0.00000300 sec
D1               1.50000000 sec
D2               0.00344828 sec
D6               0.05000000 sec
D16              0.00020000 sec
IN0              0.00001809 sec
TDav                      1
SFO1            499.8726683 MHz
NUC1                     1H
P1                     9.73 usec
P2                    19.46 usec
PLW1            32.18000031 W
SFO2            125.7049807 MHz
NUC2                    13C
P3                     9.00 usec
PLW2           106.93000031 W
GPNAM[1]         SMSQ10.100
GPZ1                  50.00 %
GPNAM[2]         SMSQ10.100
GPZ2                  30.00 %
GPNAM[3]         SMSQ10.100
GPZ3                  40.10 %
P16                 1000.00 usec

======= F1 INDIRECT DIMENSION =======
td1        128
sw_F1      220.000000

F1 - Acquisition parameters
TD                      128
SFO1                125.705 MHz
FIDRES           432.077423 Hz
SW                  219.983 ppm
FnMODE                   QF

F2 - Processing parameters
SI                     4096
SF              499.8700000 MHz
WDW                   QSINE
SSB                       0
LB                        0 Hz
GB                        0
PC                     1.40

F1 - Processing parameters
SI                     1024
MC2                      QF
SF              125.6924115 MHz
WDW                   QSINE
SSB                       0
LB                        0 Hz
GB                        0
```

AZA-ST 1c

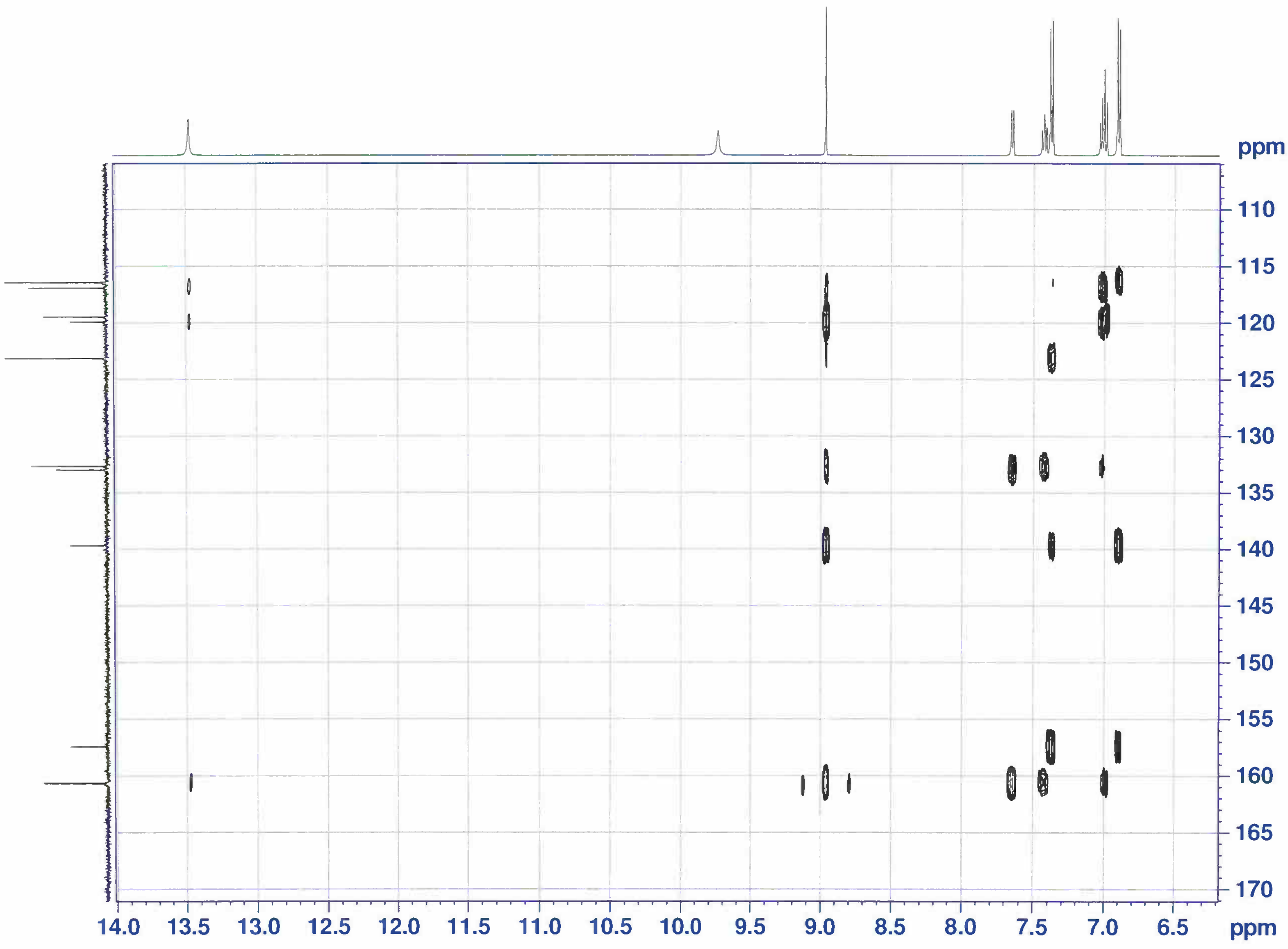

Current Data Parameters	
NAME	22ddu_AZA3
EXPNO	7
PROCNO	1

F2 - Acquisition Parameters	
Date_	20220601
Time	1.56 h
INSTRUM	Avance NEO
PROBHD	Z167419_0024 (
PULPROG	hmbcgplpndqf
TD	4096
SOLVENT	DMSO
NS	16
DS	16
SWH	7462.687 Hz
FIDRES	3.643890 Hz
AQ	0.2744320 sec
RG	101
DW	67.000 usec
DE	6.50 usec
TE	297.9 K
CNST2	145.0000000
CNST13	10.0000000
D0	0.00000300 sec
D1	1.50000000 sec
D2	0.00344828 sec
D6	0.05000000 sec
D16	0.00020000 sec
IN0	0.00001809 sec
TDav	1
SFO1	499.8736628 MHz
NUC1	1H
P1	9.73 usec
P2	19.46 usec
PLW1	32.18000031 W
SFO2	125.7049807 MHz
NUC2	13C
P3	9.00 usec
PLW2	106.93000031 W
GPNAM[1]	SMSQ10.100
GPZ1	50.00 %
GPNAM[2]	SMSQ10.100
GPZ2	30.00 %
GPNAM[3]	SMSQ10.100
GPZ3	40.10 %
P16	1000.00 usec

======= F1 INDIRECT DIMENSION =======

td1	128
sw_F1	220.000000

F1 - Acquisition parameters	
TD	128
SFO1	125.705 MHz
FIDRES	432.077423 Hz
SW	219.983 ppm
FnMODE	QF

F2 - Processing parameters	
SI	4096
SF	499.8699721 MHz
WDW	QSINE
SSB	0
LB	0 Hz
GB	0
PC	1.40

F1 - Processing parameters	
SI	1024
MC2	QF
SF	125.6924115 MHz
WDW	QSINE
SSB	0
LB	0 Hz
GB	0

A2A-ST 1d

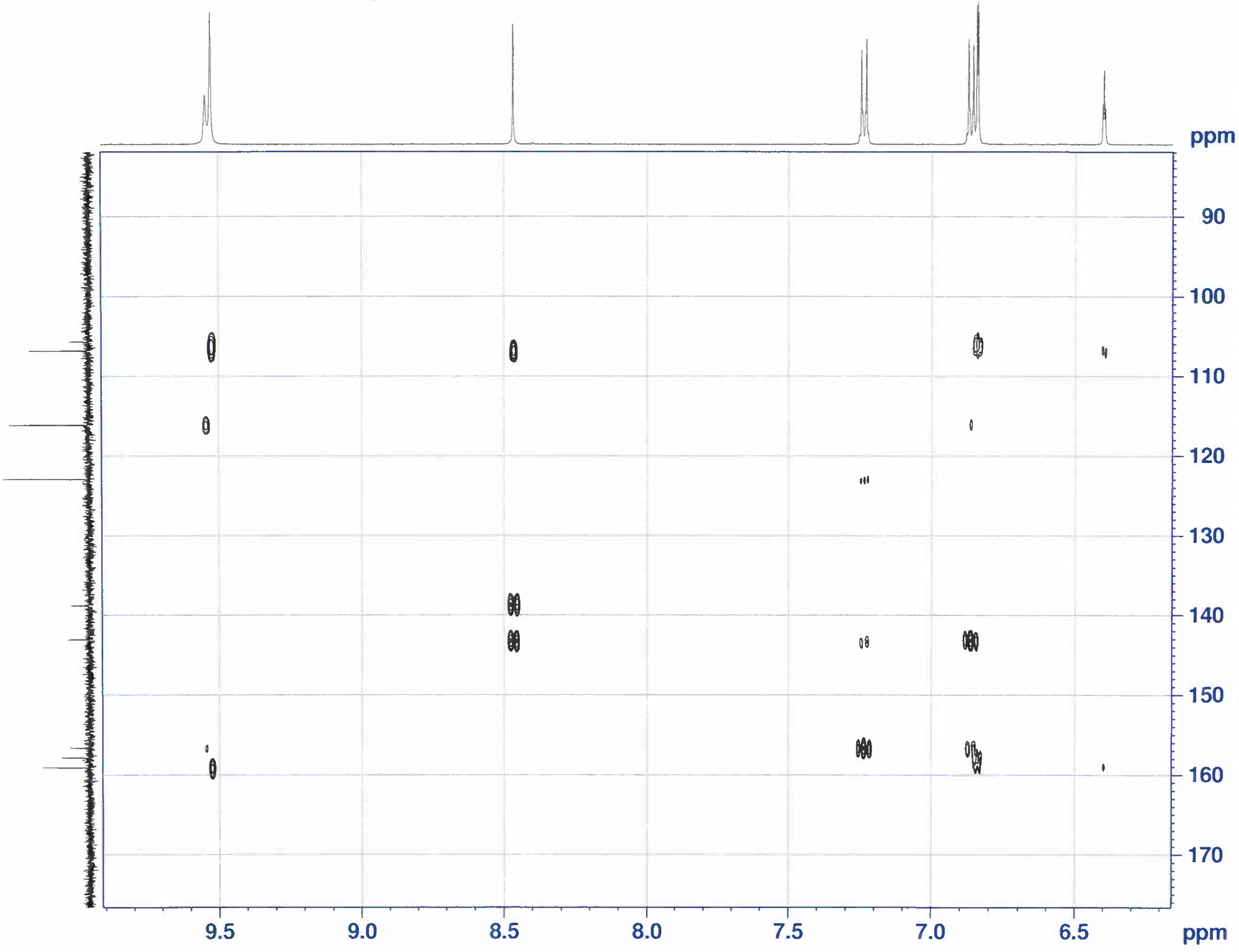

Current Data Parameters

NAME	22ddu_AZA4	
EXPNO	7	
PROCNO	1	

F2 - Acquisition Parameters

Date_	20220601	
Time	4.18	h
INSTRUM	Avance NEO	
PROBHD	Z167419_0024 (	
PULPROG	hmbcgplpndqf	
TD	4096	
SOLVENT	DMSO	
NS	16	
DS	16	
SWH	5882.353	Hz
FIDRES	2.872243	Hz
AQ	0.3481600	sec
RG	101	
DW	85.000	usec
DE	6.50	usec
TE	297.9	K
CNST2	145.0000000	
CNST13	10.0000000	
D0	0.00000300	sec
D1	1.50000000	sec
D2	0.00344828	sec
D6	0.05000000	sec
D16	0.00020000	sec
IN0	0.00001809	sec
TDav	1	
SFO1	499.8723458	MHz
NUC1	1H	
P1	9.73	usec
P2	19.46	usec
PLW1	32.18000031	W
SFO2	125.7049807	MHz
NUC2	13C	
P3	9.00	usec
PLW2	106.93000031	W
GPNAM[1]	SMSQ10.100	
GPZ1	50.00	%
GPNAM[2]	SMSQ10.100	
GPZ2	30.00	%
GPNAM[3]	SMSQ10.100	
GPZ3	40.10	%
P16	1000.00	usec

======== F1 INDIRECT DIMENSION ========

td1	128	
sw_F1	220.000000	

F1 - Acquisition parameters

TD	128	
SFO1	125.705	MHz
FIDRES	432.077423	Hz
SW	219.983	ppm
FnMODE	QF	

F2 - Processing parameters

SI	4096	
SF	499.8699593	MHz
WDW	QSINE	
SSB	0	
LB	0	Hz
GB	0	
PC	1.40	

F1 - Processing parameters

SI	1024	
MC2	QF	
SF	125.6924115	MHz
WDW	QSINE	
SSB	0	
LB	0	Hz
GB	0	

AZA-ST 1e

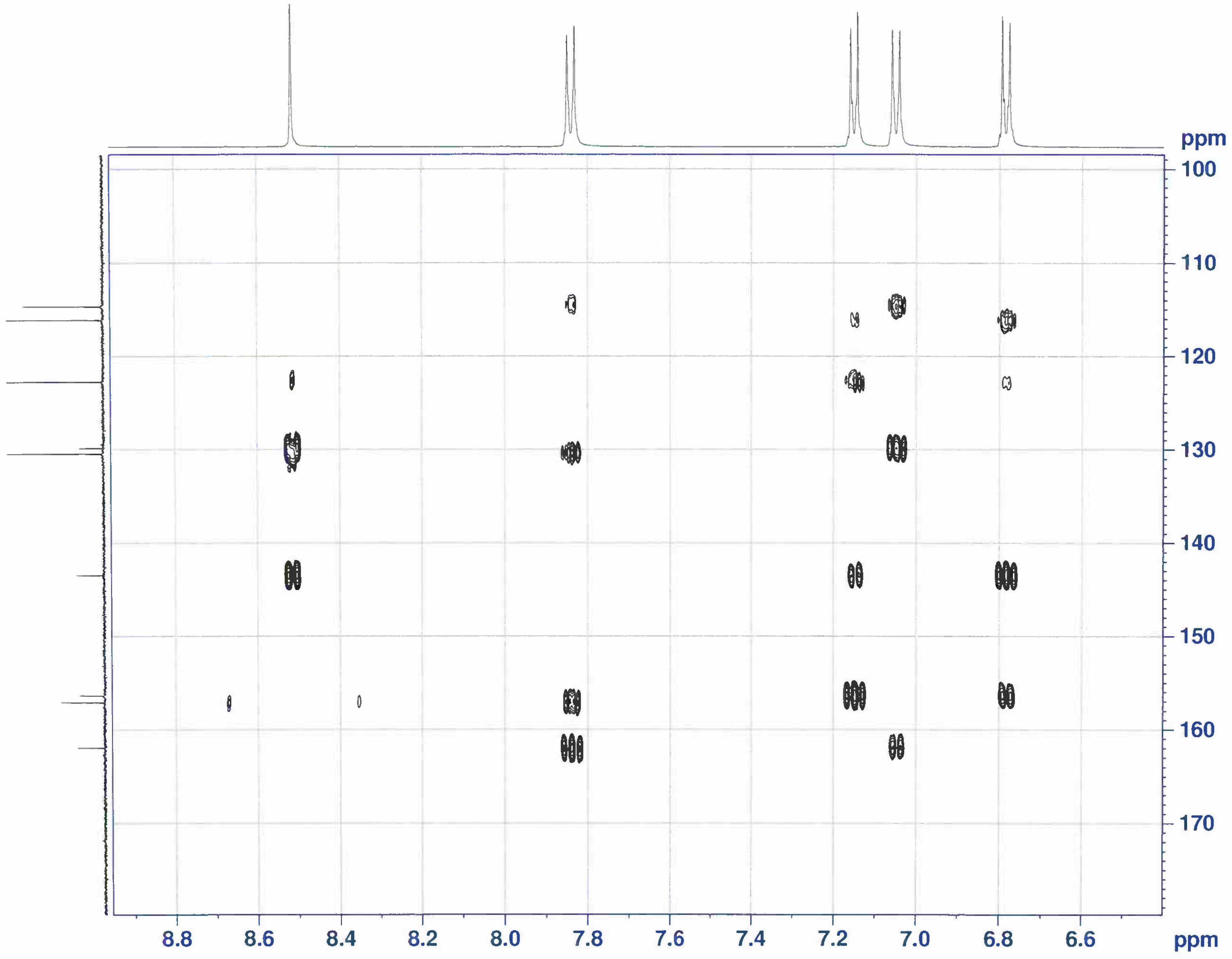

Current Data Parameters
NAME 22ddu_AZA5
EXPNO 7
PROCNO 1

F2 - Acquisition Parameters
Date_ 20220601
Time 6.41 h
INSTRUM Avance NEO
PROBHD Z167419_0024 (
PULPROG hmbcgplpndqf
TD 4096
SOLVENT DMSO
NS 16
DS 16
SWH 4166.667 Hz
FIDRES 2.034505 Hz
AQ 0.4915200 sec
RG 101
DW 120.000 usec
DE 6.50 usec
TE 297.9 K
CNST2 145.0000000
CNST13 10.0000000
D0 0.00000300 sec
D1 1.50000000 sec
D2 0.00344828 sec
D6 0.05000000 sec
D16 0.00020000 sec
IN0 0.00001809 sec
TDav 1
SFO1 499.8729821 MHz
NUC1 1H
P1 9.73 usec
P2 19.46 usec
PLW1 32.18000031 W
SFO2 125.7049807 MHz
NUC2 13C
P3 9.00 usec
PLW2 106.93000031 W
GPNAM[1] SMSQ10.100
GPZ1 50.00 %
GPNAM[2] SMSQ10.100
GPZ2 30.00 %
GPNAM[3] SMSQ10.100
GPZ3 40.10 %
P16 1000.00 usec

======== F1 INDIRECT DIMENSION ========
td1 128
sw_F1 220.000000

F1 - Acquisition parameters
TD 128
SFO1 125.705 MHz
FIDRES 432.077423 Hz
SW 219.983 ppm
FnMODE QF

F2 - Processing parameters
SI 4096
SF 499.8700000 MHz
WDW QSINE
SSB 0
LB 0 Hz
GB 0
PC 1.40

F1 - Processing parameters
SI 1024
MC2 QF
SF 125.6924115 MHz
WDW QSINE
SSB 0
LB 0 Hz
GB 0

AZA-ST 1f

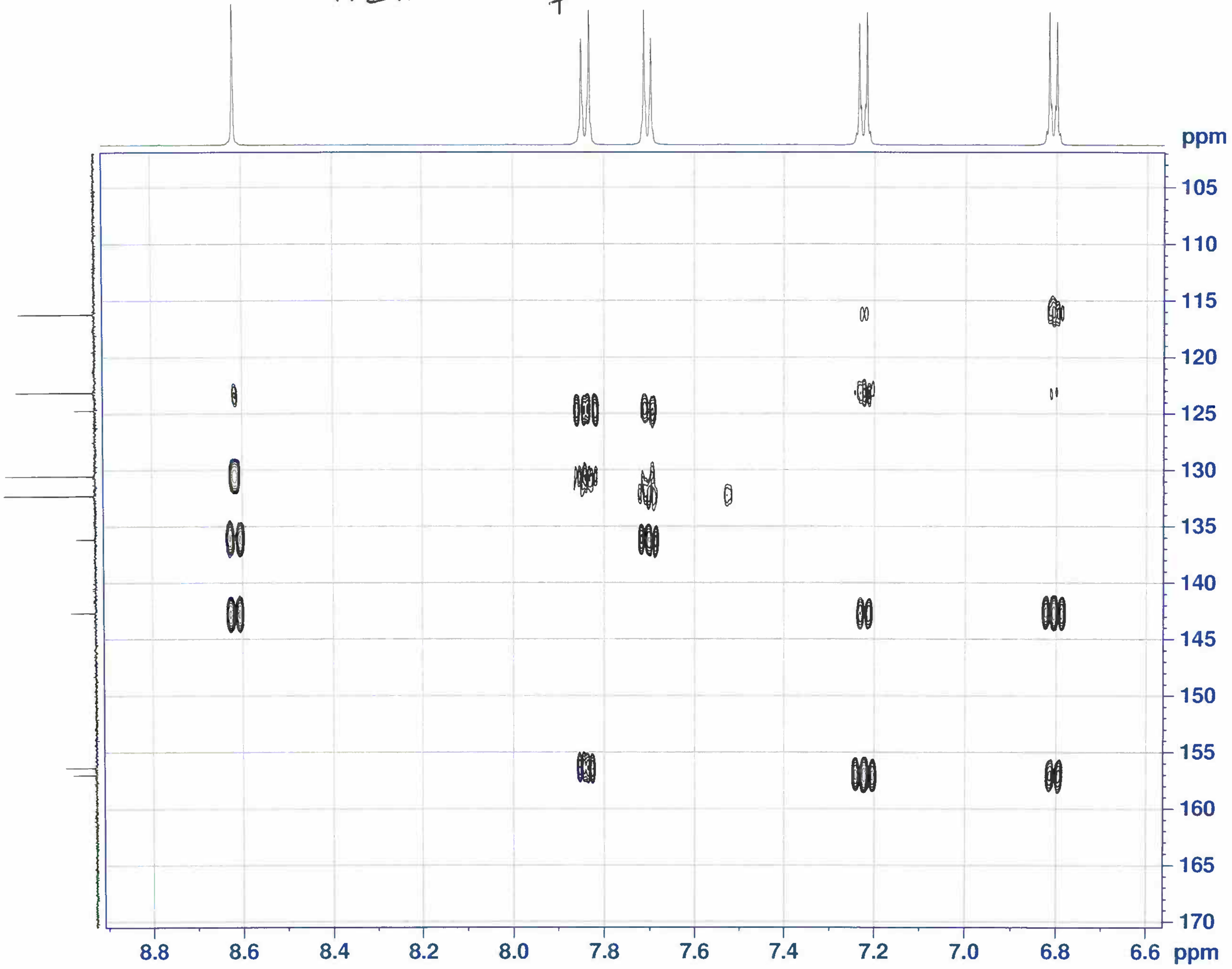


```
Current Data Parameters
NAME           22ddu_AZA6
EXPNO                   7
PROCNO                  1

F2 - Acquisition Parameters
Date_            20220601
Time                21.15 h
INSTRUM        Avance NEO
PROBHD     Z167419_0024 (
PULPROG     hmbcgplpndqf
TD                   4096
SOLVENT              DMSO
NS                     16
DS                     16
SWH              4545.455 Hz
FIDRES           2.219460 Hz
AQ              0.4505600 sec
RG                    101
DW                110.000 usec
DE                   6.50 usec
TE                  297.8 K
CNST2         145.0000000
CNST13         10.0000000
D0             0.00000300 sec
D1             1.50000000 sec
D2             0.00344828 sec
D6             0.05000000 sec
D16            0.00020000 sec
IN0            0.00001809 sec
TDav                    1
SFO1          499.8729068 MHz
NUC1                   1H
P1                   9.73 usec
P2                  19.46 usec
PLW1          32.18000031 W
SFO2          125.7049807 MHz
NUC2                  13C
P3                   9.00 usec
PLW2         106.93000031 W
GPNAM[1]       SMSQ10.100
GPZ1                50.00 %
GPNAM[2]       SMSQ10.100
GPZ2                30.00 %
GPNAM[3]       SMSQ10.100
GPZ3                40.10 %
P16               1000.00 usec

======== F1 INDIRECT DIMENSION ========
td1         128
sw_F1       220.000000

F1 - Acquisition parameters
TD                    128
SFO1              125.705 MHz
FIDRES         432.077423 Hz
SW                219.983 ppm
FnMODE                 QF

F2 - Processing parameters
SI                   4096
SF            499.8700000 MHz
WDW                 QSINE
SSB                     0
LB                      0 Hz
GB                      0
PC                   1.40

F1 - Processing parameters
SI                   1024
MC2                    QF
SF            125.6924115 MHz
WDW                 QSINE
SSB                     0
LB                      0 Hz
GB                      0
```

AZA-ST 1g

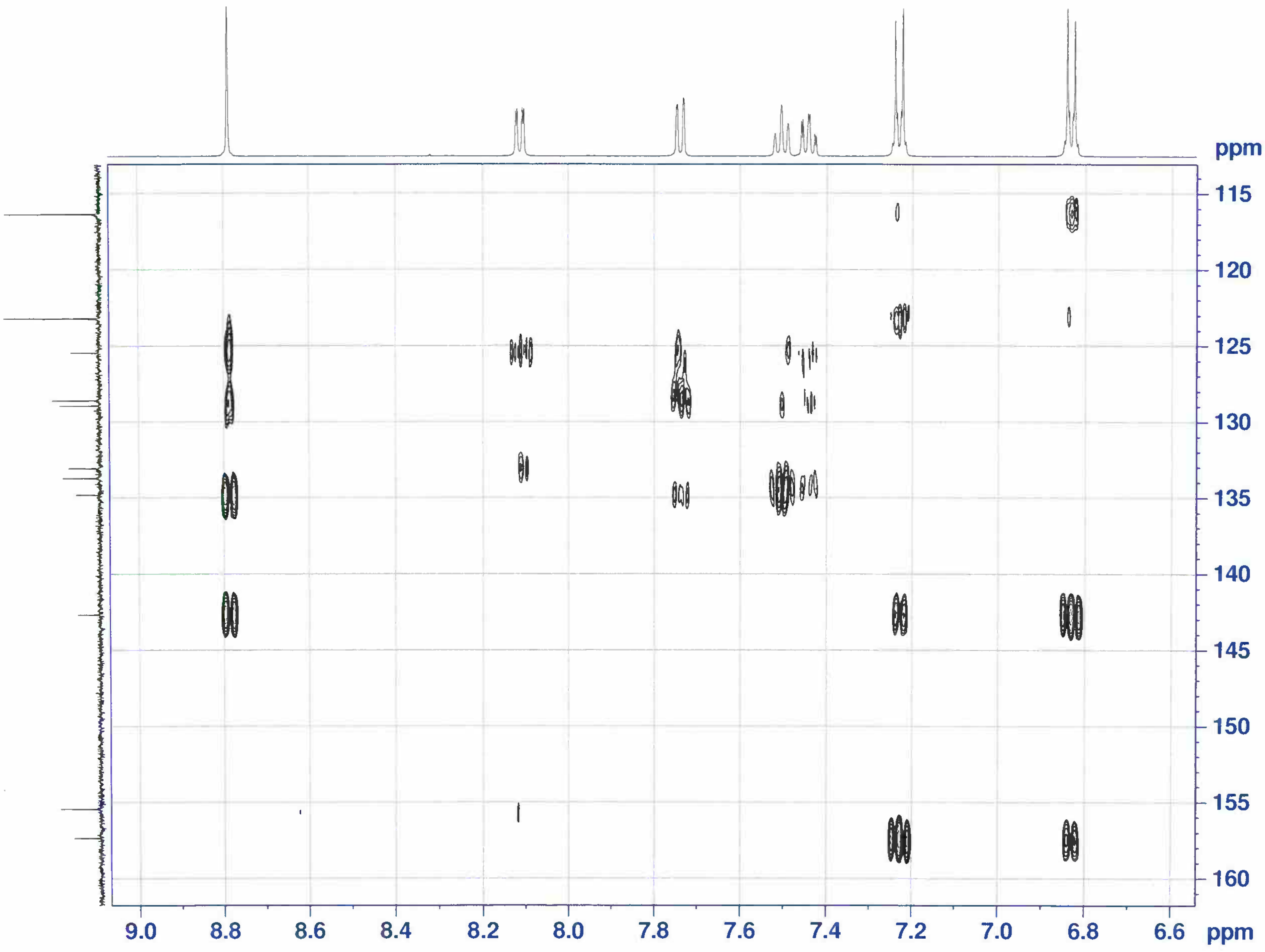

Current Data Parameters

NAME	22ddu_AZA7
EXPNO	7
PROCNO	1

F2 - Acquisition Parameters

Date_	20220601
Time	23.37 h
INSTRUM	Avance NEO
PROBHD	Z167419_0024 (
PULPROG	hmbcgplpndqf
TD	4096
SOLVENT	DMSO
NS	16
DS	16
SWH	4310.345 Hz
FIDRES	2.104661 Hz
AQ	0.4751360 sec
RG	101
DW	116.000 usec
DE	6.50 usec
TE	297.9 K
CNST2	145.0000000
CNST13	10.0000000
D0	0.00000300 sec
D1	1.50000000 sec
D2	0.00344828 sec
D6	0.05000000 sec
D16	0.00020000 sec
IN0	0.00001809 sec
TDav	1
SFO1	499.8730295 MHz
NUC1	1H
P1	9.73 usec
P2	19.46 usec
PLW1	32.18000031 W
SFO2	125.7049807 MHz
NUC2	13C
P3	9.00 usec
PLW2	106.93000031 W
GPNAM[1]	SMSQ10.100
GPZ1	50.00 %
GPNAM[2]	SMSQ10.100
GPZ2	30.00 %
GPNAM[3]	SMSQ10.100
GPZ3	40.10 %
P16	1000.00 usec

======== F1 INDIRECT DIMENSION ========

td1	128
sw_F1	220.000000

F1 - Acquisition parameters

TD	128
SFO1	125.705 MHz
FIDRES	432.077423 Hz
SW	219.983 ppm
FnMODE	QF

F2 - Processing parameters

SI	4096
SF	499.8700000 MHz
WDW	QSINE
SSB	0
LB	0 Hz
GB	0
PC	1.40

F1 - Processing parameters

SI	1024
MC2	QF
SF	125.6924115 MHz
WDW	QSINE
SSB	0
LB	0 Hz
GB	0

Resveratrol
Aza-Stilbene 1a
Aza-Stilbene 1b
Aza-Stilbene 1c
Aza-Stilbene 1d
Aza-Stilbene 1e
Aza-Stilbene 1f
Aza-Stilbene 1g

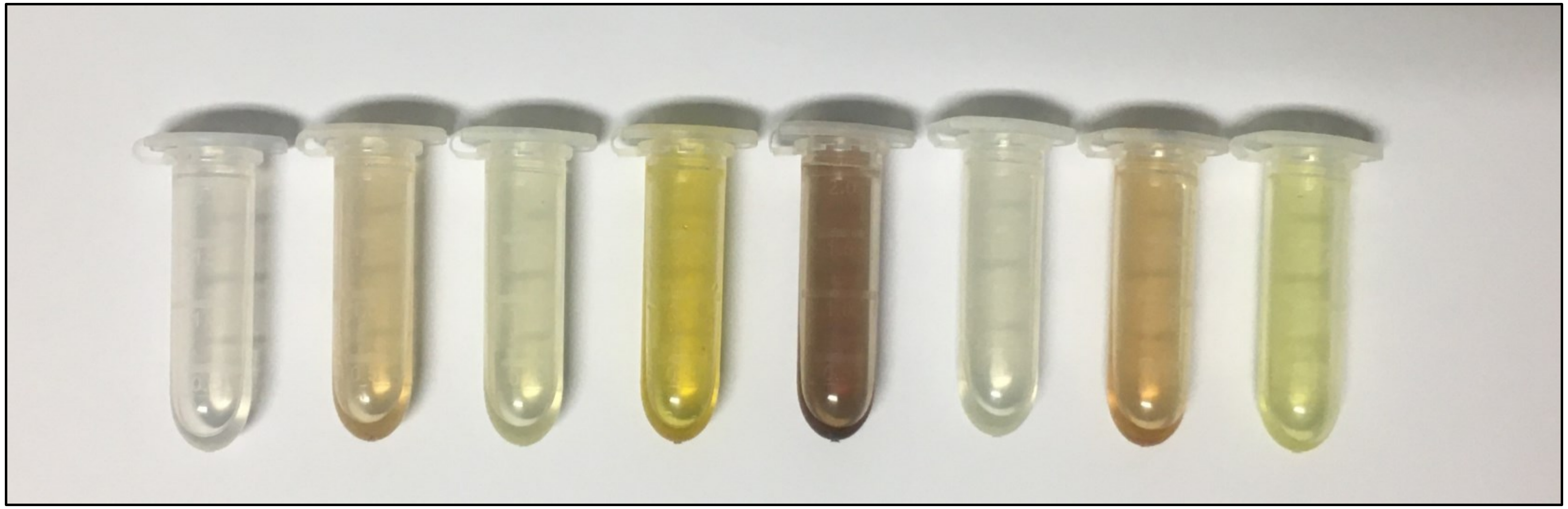

- Stock solutions were prepared at 50 mM
- Resveratrol (*trans*-resveratrol) was diluted in absolute ethanol (EtOH)
- Aza-Stilbenes 1a to 1g were diluted in dimethyl sulfoxide (DMSO)

Supplementary Figure S3: Ksila M et al.
